# Enthalpy‐Driven Molecular Engineering Enables High‐Performance Quasi‐Solid‐State Electrolytes for Long Life Lithium Metal Batteries

**DOI:** 10.1002/adma.202419335

**Published:** 2025-04-07

**Authors:** Zilong Wang, Longyun Shen, Yilin Ma, Ho Mei Law, Shengjun Xu, Yixin Bi, Matthew J. Robson, Yuhao Wang, André Gröschel, Qing Chen, Francesco Ciucci

**Affiliations:** ^1^ Department of Mechanical and Aerospace Engineering The Hong Kong University of Science and Technology Clear Water Bay, Kowloon Hong Kong SAR 999077 China; ^2^ Division of Emerging Interdisciplinary Areas The Hong Kong University of Science and Technology Clear Water Bay, Kowloon Hong Kong SAR 999077 China; ^3^ Chair of Electrode Design for Electrochemical Energy Systems University of Bayreuth Weiherstraße 26 95448 Bayreuth Germany; ^4^ Bavarian Center for Battery Technology (BayBatt) University of Bayreuth Universitätsstraße 30 95447 Bayreuth Germany; ^5^ Polymer Materials for Energy Storage (PES) and Macromolecular Chemistry University of Bayreuth Weiherstraße 26 95448 Bayreuth Germany

**Keywords:** in situ polymerization, Li‐metal battery, polymerization enthalpy, pouch‐type battery, quasi‐solid‐state electrolyte

## Abstract

The advancement of lithium metal batteries toward their theoretical energy density potential remains constrained by safety and performance issues inherent to liquid electrolytes. Quasi‐solid‐state electrolytes (QSSEs) based on poly‐1,3‐dioxolane (poly‐DOL) represent a promising development, yet challenges in achieving satisfactory Coulombic efficiency and long‐term stability have impeded their practical implementation. While lithium nitrate addition can enhance efficiency, its incorporation results in prohibitively slow polymerization rates spanning several months. In this work, high‐polymerization‐enthalpy 1,1,1‐trifluoro‐2,3‐epoxypropane is introduced as a co‐polymerization promoter, successfully integrating lithium nitrate into poly‐DOL‐based QSSEs. The resulting electrolyte demonstrates exceptional performance with 2.23 mS cm^−1^ of ionic conductivity at 25 °C, a Coulombic efficiency of 99.34% in Li|Cu cells, and stable lithium metal interfaces sustained through 1300 h of symmetric cell cycling. This co‐polymerization approach also suppresses poly‐DOL crystallization, enabling Li|LiFePO_4_ cells to maintain stability beyond 2000 cycles at 1C. Scale‐up validation in a ≈1 Ah Li|NCM811 pouch cell achieves 94.4% capacity retention over 60 cycles. This strategy establishes a new pathway for developing high‐performance, in situ polymerized quasi‐solid‐state batteries for practical energy storage applications.

## Graphical abstract

This work introduces a co‐polymerization approach using 1,1,1‐trifluoro‐2,3‐epoxypropane to integrate lithium nitrate into poly‐DOL‐based quasi‐solid‐state electrolytes. The resulting electrolyte exhibits high ionic conductivity (2.23 mS cm^−1^), exceptional Coulombic efficiency (99.34%) in Li|Cu cell, and long‐term stability (2000 cycles in Li|LFP cells). Scale‐up tests in Li|NCM811 cells show 94.4% capacity retention over 60 cycles, offering a promising path for high‐performance, in situ polymerized batteries.

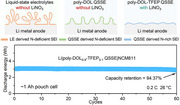

## 1 Introduction

The search for high energy‐density batteries has established lithium metal as a critical next‐generation anode material, offering theoretical capacities far exceeding those achievable by the current lithium‐ion system.^[^ ^1^ ^]^ However, the commercial adoption of Li‐metal technology continues to be hindered by lithium dendrite formation, interfacial instability, and safety concerns.^[^ ^2^ ^]^ While solid‐state electrolytes (SSEs) have been extensively explored as alternatives,^[^ ^3^ ^]^ challenges from ionic conductivity, interfacial contact resistances, and scalability have impeded their practical implementation.^[^ ^4^ ^]^ To bridge this gap, in situ polymerized quasi‐solid‐state electrolytes (QSSEs) have surfaced as a promising route toward safe, high‐energy‐density, and commercially feasible lithium metal batteries.^[^ ^4^, ^5^ ^]^

The synthesis of QSSEs through in situ polymerization involves the injection of a liquid precursor solution into a cell, followed by its polymerization. This approach produces quasi‐solid‐state batteries that outperform both ceramic and conventional polymer batteries due to improved interfacial contacts and unobstructed lithium ion pathways.^[^ ^6^ ^]^ Among the various options, poly‐1,3‐dioxolane (poly‐DOL) QSSEs, synthesized through ring‐opening polymerization (ROP) of 1,3‐dioxolane (DOL), have emerged as a particularly promising candidate.^[^ ^7^ ^]^ Poly‐DOL QSSEs exhibit several advantageous properties, including high ionic conductivity exceeding 1 mS cm^−1^ at room temperature, excellent stability against lithium metal, and superior oxidative stability surpassing 5.0 V *vs*. Li^+^/Li for pure poly‐DOL.^[^ ^8^ ^]^ Despite their advantages over liquid electrolytes, poly‐DOL‐based QSSEs still struggle with long‐term cycling stability due to suboptimal Coulombic efficiencies (CEs) and crystallization that limits Li⁺ transport.

Lithium nitrate (LiNO_3_) enhances CE by forming a nitrogen‐rich solid electrolyte interphase (SEI) on lithium metal anodes (LMAs).^[^ ^9^ ^]^ This Li_3_N/Li_x_N_y_‐rich layer promotes uniform Li^+^ deposition and surface stability while suppressing parasitic reactions.^[^ ^9^, ^10^ ^]^ Incorporating LiNO_3_ into poly‐DOL‐based QSSEs poses a fundamental challenge because it inhibits the ROP of DOL, which is a crucial step in poly‐DOL formation.^[^ ^5^, ^11^ ^]^ This incompatibility has limited the development of stable, high‐performance quasi‐solid‐state lithium metal batteries.^[^ ^11b^ ^]^ Notably, Archer *et al*. demonstrated that the inhibitory effect of LiNO_3_ is most pronounced during the initiation period of the ROP process,^[^ ^12^ ^]^ suggesting that overcoming this initial barrier could enable the integration of both LiNO_3_ and poly‐DOL‐based QSSEs.

**Figure 1:**
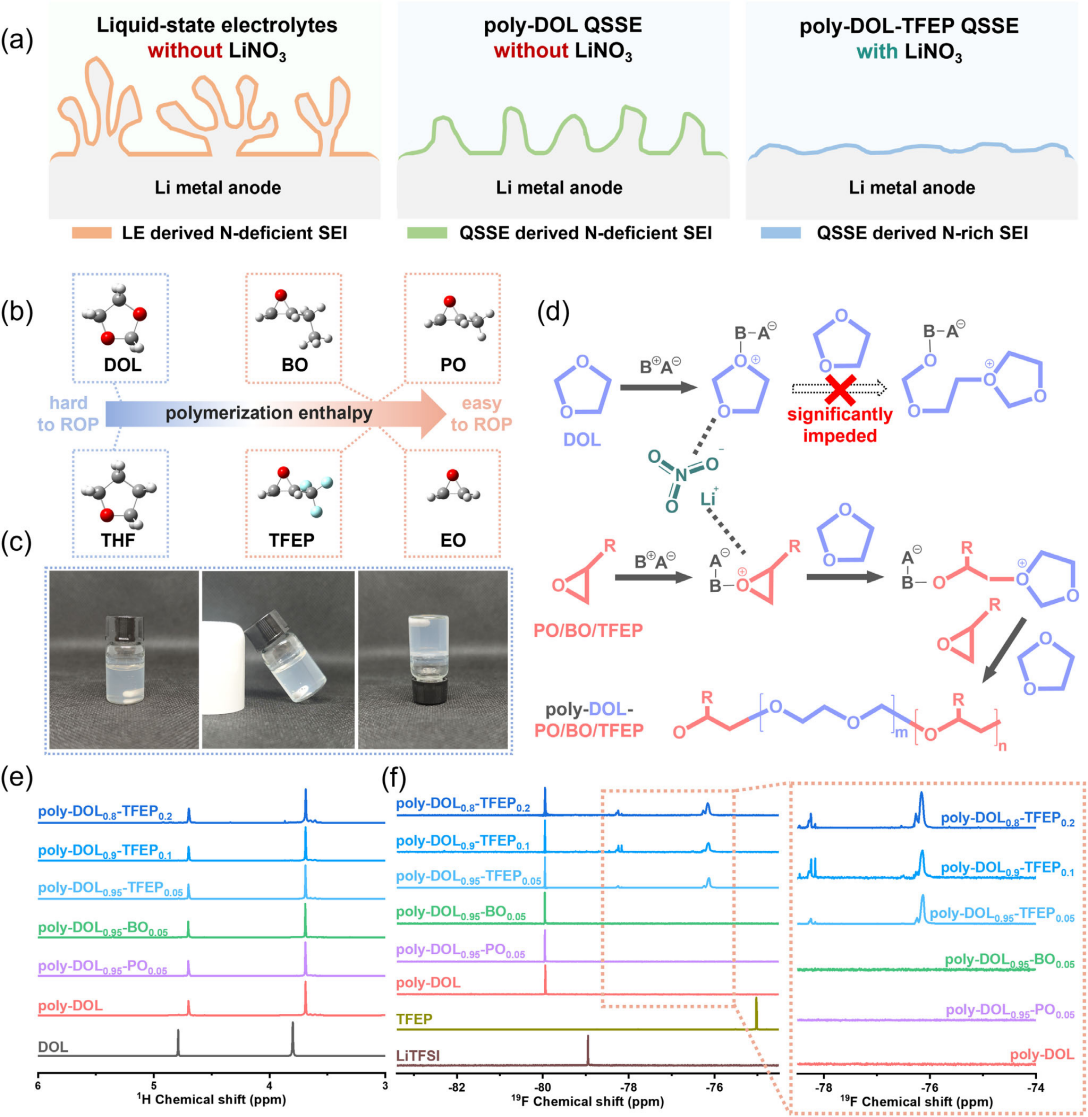
Electrolyte design and polymerization mechanism. a) Schematic representation of the evolution of the Li metal anode (LMA) surface: dendrites formation in the liquid electrolyte (LE) without LiNO_3_ (left), reduced dendrites formation in poly‐DOL QSSE without LiNO_3_ (middle), and a smooth surface achieved through the combination of LiNO_3_‐derived N‐rich SEI and poly‐DOL QSSE (right). b) Comparison of polymerization enthalpies: three‐membered cyclic ethers (EO, PO, BO, TFEP) exhibit higher values and facilitate easier ring‐opening polymerization (ROP) compared to their five‐membered counterparts (THF, DOL). c) Representative poly‐DOL_0.9_‐TFEP_0.1_ QSSE. d) Polymerization mechanism: while LiNO_3_ inhibits DOL polymerization, high‐enthalpy three‐membered cyclic ethers form tertiary oxonium ions even in the presence of LiNO_3_, which induces and accelerates DOL's ROP (as supported by Figures S6 and S7, Supporting Information) (‐R represents ─CH_3_ (PO), ─CH_2_CH_3_ (BO), or ─CF_3_ (TFEP)). e) ^1^H NMR spectra comparing the DOL monomer and various poly‐DOL‐based QSSE polymer backbones. f) ^19^F NMR spectra of LiTFSI, TFEP, and poly‐DOL‐based QSSE polymer backbones.

Herein, we present an enthalpy‐driven molecular engineering approach that addresses the incompatibility between LiNO_3_ and poly‐DOL‐based QSSEs by introducing 1,1,1‐trifluoro‐2,3‐epoxypropane (TFEP), a cyclic ether with high polymerization enthalpy, as a co‐polymerization promoter. The resulting system integrates LiNO_3_‐derived nitrogen‐rich SEI with QSSE protection, enabling uniform Li⁺ deposition and dendrite suppression (**Figure** **1**a) while outperforming conventional liquid electrolytes in both stability and efficiency. The TFEP co‐polymerization also suppresses crystallization, enhancing ionic conductivity and long‐term performance. In Li|LiFePO_4_ coin cells, the LiNO_3_‐containing poly‐DOL_0.9_‐TFEP_0.1_ QSSE (DOL: TFEP = 0.9: 0.1, mass ratio) can cycle stably for over 2,000 times at 1C, significantly exceeding the 500 cycles of corresponding liquid electrolyte cells. Notably, a ≈1 Ah Li|NCM811(LiNi_0.8_Co_0.1_Mn_0.1_O_2_) pouch cell utilizing the poly‐DOL_0.9_‐TFEP_0.1_ QSSE achieved an initial specific energy of 302 Wh kg^−1^ while maintaining 94.4% of its initial capacity after 60 cycles. This molecular engineering strategy based on polymerization enthalpy opens new avenues for the development of quasi‐solid‐state batteries with superior capacity and stability.

## 2 Results and Discussion

### 2.1 Incorporating LiNO_3_ into Poly‐DOL‐Based QSSEs with an Enthalpy‐Driven Co‐Polymerization Strategy

Lewis acids (e.g., BF_3_^−^,^[^ ^13^ ^]^ PF_5_^−^,^[^ ^8b^ ^]^ (otf)_3_^−^,^[^ ^8d^ ^]^ etc.^[^ ^8^, ^14^ ^]^) initiate the ROP of DOL by generating oxonium ions, which then drive chain growth through sequential monomer addition. The process evolves from the initial coordination of DOL and the initiator to chain propagation via continuous oxonium ion formation (see Figure S1, Supporting Information).^[^ ^11^, ^15^ ^]^ While LiNO_3_ enhances battery CE by forming Li_3_N/Li_x_N_y_‐rich SEI on LMA,^[^ ^9^, ^10^ ^]^ it critically inhibits DOL's ROP.^[^ ^5^, ^11^, ^12^ ^]^ In fact, DOL fails to polymerize for over five months at room temperature when LiNO_3_ is present (Table S2, Supporting Information). Given DOL's low boiling point (74 °C), alternative room‐temperature strategies are needed to overcome this polymerization barrier.

The relationship between ring strain and polymerization enthalpy guided our approach to DOL polymerization, drawing inspiration from ethylene oxide (EO) promotion in polytetrahydrofuran (poly‐THF) synthesis. The polymerization enthalpy (Δ*H*_poly_) serves as the primary driving force in ROP of cyclic ethers, reflecting the energy difference between polymeric products and monomeric reactants. This differential correlates directly with the ring strain of the monomer^[^ ^15^, ^16^ ^]^ (see Table S1 and Figure S2, Supporting Information for further details). The three‐membered cyclic structure of EO exhibits a significantly higher polymerization enthalpy than THF (Table S1, Supporting Information), resulting in increased ring‐opening propensity during ROP initiation.^[^ ^17^ ^]^ This characteristic enables EO to function as an effective promoter in THF polymerization through rapid ring‐opening and tertiary oxonium ion generation. These ions subsequently induce THF polymerization (further details are provided in Figure S3, Supporting Information),^[^ ^11^, ^15^, ^16^, ^18^ ^]^ which addresses the slow initiation phase of THF ROP and accelerates the overall poly‐THF synthesis process. In this article, we hypothesize that three‐membered cyclic ethers can facilitate DOL polymerization despite the presence of the ROP inhibitor LiNO_3_. Given the impracticality of EO (boiling point 10.7 °C) for room‐temperature synthesis, we selected three alternative promoters with higher boiling points and similar polymerization enthalpies (Figure 1b; Table S1, Supporting Information): propylene oxide (PO) (Figure S4a, Supporting Information), 1,2‐butylene oxide (BO) (Figure S4b, Supporting Information), and 1,1,1‐trifluoro‐2,3‐epoxypropane (TFEP) (Figure S4c,d, Supporting Information). Experimental validation confirmed our hypothesis, as introducing just 5 wt.% PO or BO enabled DOL polymerization within 48 h, even in systems containing 0.2 m LiNO_3_ (Figure 1c; Table S2 and Figure S5, Supporting Information). The mechanism underlying this process is illustrated in Figure 1d.

Fourier‐transform infrared (FTIR) spectroscopy and nuclear magnetic resonance (NMR) spectroscopy were used to characterize the prepared electrolytes (Figure S10, Supporting Information; Figure 1e,f). Throughout this study, unless otherwise specified, the poly‐DOL QSSE contained no LiNO_3_, whereas all co‐polymerized poly‐DOL‐based QSSEs (poly‐DOL_1−x_‐PO_x_ QSSE, poly‐DOL_1−x_‐BO_x_, QSSE, and poly‐DOL_1−x_‐TFEP_x_ QSSE) incorporated 0.2 m LiNO_3_. FTIR spectra revealed a characteristic peak at 844.7 cm^−1^ corresponding to ─(CH_2_)_n_─, indicating polymer chain formation (Figure S10, Supporting Information). For polymer backbone structure analysis, residual liquid components were removed through ethanol centrifugation before NMR analysis (Figure 1e,f). The ^1^H NMR spectra of poly‐DOL‐based QSSEs exhibited distinctive upfield shifts relative to monomeric DOL, with characteristic signals moving from 4.79 ppm and 3.80 ppm to 4.70 ppm and 3.69 ppm, respectively, confirming successful polymerization.^[^ ^8b,d^ ^]^ Furthermore, within the TFEP‐containing QSSEs, the ^19^F NMR spectrum displayed peaks at −76.10 and −78.20 ppm (Figure 1f) attributable to the TFEP‐derived ─CF_3_ group, suggesting the successful co‐polymerization of TFEP and DOL.^[^ ^19^ ^]^

### 2.2 LiNO_3_‐Containing Poly‐DOL‐Based QSSEs

**Figure 2:**
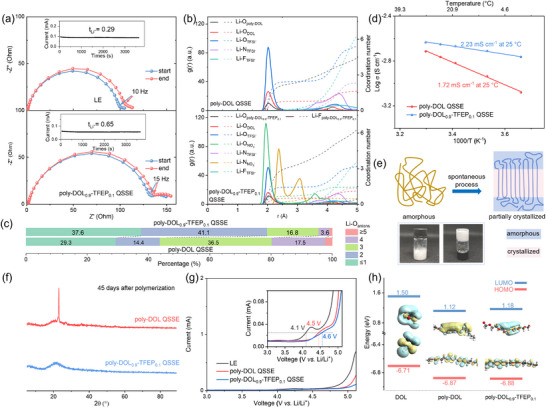
Electrochemical and structural analysis of electrolytes. a) Electrochemical impedance spectra and chronoamperometry data for Li symmetric cells containing LE (top) and poly‐DOL_0.9_‐TFEP_0.1_ QSSE (bottom). b) Radial distribution functions and coordination numbers for Li‐atom pairs in poly‐DOL QSSE (upper) and poly‐DOL_0.9_‐TFEP_0.1_ QSSE (lower). c) Distribution of Li^+^ solvation structures among various anions in poly‐DOL QSSE and poly‐DOL_0.9_‐TFEP_0.1_ QSSE. d) Li^+^ conductivity as a function of temperature for poly‐DOL QSSE and poly‐DOL_0.9_‐TFEP_0.1_ QSSE. e) Schematic representation of spontaneous room‐temperature crystallization in the poly‐DOL backbone, with inset photographs showing crystallized poly‐DOL (white‐colored substance) at 45 days post‐polymerization. f) XRD patterns for poly‐DOL QSSE and poly‐DOL_0.9_‐TFEP_0.1_ QSSE at 45 days post‐polymerization. g) LSV profiles for LE, poly‐DOL QSSE, and poly‐DOL_0.9_‐TFEP_0.1_ QSSE. h) HOMO/LUMO energy levels for the DOL monomer, poly‐DOL backbone, and poly‐DOL_0.9_‐TFEP_0.1_ backbone.

The Li^+^ transference numbers of the synthesized poly‐DOL, LiNO_3_‐containing co‐polymerized poly‐DOL‐based QSSEs, and LE (1 m LiTFSI in DOL) were evaluated using Li symmetrical cells (**Figure** **2**a; Figure S11, Supporting Information). The analysis revealed Li^+^ transference numbers of 0.59, 0.65, 0.67, 0.71, and 0.65 for poly‐DOL QSSE, poly‐DOL_0.95_‐PO_0.05_ QSSE, poly‐DOL_0.95_‐BO_0.05_ QSSE, poly‐DOL_0.95_‐TFEP_0.05_ QSSE, and poly‐DOL_0.9_‐TFEP_0.1_ QSSE, respectively. Notably, all QSSEs exhibited substantially higher transference numbers than the LE (0.29). This enhancement can be attributed to the strong coordination between anionic species and polymer chains within the QSSEs and is consistent with previous studies.^[^ ^8^, ^20^ ^]^ Thermogravimetric analysis (TGA) was used to assess the mass loss of the LE and the synthesized QSSEs as a function of temperature (Figure S12, Supporting Information). The low boiling point (74 °C) of DOL renders the LE susceptible to weight loss even at room temperature (Figure S12a, Supporting Information). Conversely, the TGA profiles of the poly‐DOL‐based QSSEs exhibited enhanced thermal stability compared to the LE (Figure S12b–e, Supporting Information).

The dissociation of lithium salt in QSSEs is directly related to anion adsorption on the polymer backbone, with increased dissociation leading to higher charge carrier concentration and improved ionic conductivity.^[^ ^21^ ^]^ Raman spectroscopy quantified lithium salt dissociation in both electrolyte systems, showing that the poly‐DOL_0.9_‐TFEP_0.1_ QSSE has a higher proportion of uncoordinated TFSI⁻ anions (42%) compared to the poly‐DOL QSSE (25%) (Figure S13, Supporting Information). Density functional theory (DFT) and molecular dynamics (MD) simulations illuminated the mechanism underlying this enhanced dissociation. DFT calculations (Figures S14 and S15, Supporting Information) indicated that the binding energy between the poly‐DOL_0.9_‐TFEP_0.1_ backbone and TFSI⁻ (−1.30 eV) significantly exceeds that between the poly‐DOL backbone and TFSI⁻ (−0.79 eV). This stronger polymer‐TFSI⁻ interaction in poly‐DOL_0.9_‐TFEP_0.1_ QSSE promotes enhanced salt dissociation.

MD simulations were conducted for both poly‐DOL QSSE and poly‐DOL_0.9_‐TFEP_0.1_ QSSE (Figure 2b). The analysis revealed a lower average coordination number between Li⁺ and anions in poly‐DOL_0.9_‐TFEP_0.1_ QSSE (2.04) compared to poly‐DOL QSSE (2.47), indicating enhanced lithium salt dissociation in the former. Radial distribution function analysis (Figure 2b) revealed that NO_3_^−^ anions in the poly‐DOL_0.9_‐TFEP_0.1_ QSSE readily incorporate into the first solvation shell of Li^+^, promoting NO_3_^−^ decomposition at the lithium anode interface and generating a nitrogen‐rich SEI.^[^ ^22^ ^]^ Further examination of the Li⁺ solvation shell (Figure 2c) showed a higher proportion of Li⁺ coordinated with two or fewer anions in poly‐DOL_0.9_‐TFEP_0.1_ QSSE (78.7%) compared to poly‐DOL QSSE (43.7%). This analysis, along with insights from DFT simulations and Raman analysis, supports the improved lithium salt dissociation in poly‐DOL_0.9_‐TFEP_0.1_ QSSE.^[^ ^7^, ^21^ ^]^ The ionic conductivities of the poly‐DOL‐based QSSEs were also measured (Figure 2d; Figures S16 and S17, Supporting Information). Among the synthesized QSSEs, the poly‐DOL_0.9_‐TFEP_0.1_ QSSE displayed the highest ionic conductivity (2.23 mS cm^−1^ at 25 °C), while the Li⁺ conductivity of poly‐DOL QSSE was the lowest (1.72 mS cm^−1^ at 25 °C). Consequently, poly‐DOL_0.9_‐TFEP_0.1_ QSSE was chosen for further investigation.

The ionic conduction mechanism in polymeric systems is inherently influenced by their phase characteristics. Within the amorphous phase, polymer chains perform segmental motions at temperatures above the glass transition temperature (T_g_), enabling ionic transport. However, these movements are severely constrained in crystalline regions, resulting in diminished ionic conductivity, as demonstrated in Figure 2e. The polymer backbone structure plays a decisive role in determining crystallization behavior.^[^ ^11^, ^23^ ^]^ Poly‐DOL provides a clear example of this phenomenon, where its regular arrangement of alternating ─CH_2_─CH_2_─ and ─CH_2_─O─ units promotes crystallization at room temperature. Digital images in Figure 2e indicate crystallization, which compromises the material's conductivity.^[^ ^24^ ^]^ Previous research has established that crystallization can be inhibited by introducing heteroatoms or external groups into the polymer backbone.^[^ ^25^ ^]^ Based on this principle, we hypothesized that incorporating fluorine through TFEP integration into the poly‐DOL backbone would disrupt chain regularity and symmetry, thereby reducing crystallinity. Our experimental results confirm this hypothesis. The poly‐DOL_0.9_‐TFEP_0.1_ backbone had superior resistance to crystallization compared to the unmodified poly‐DOL backbone. XRD analysis (Figure 2f) reveals a characteristic crystallization peak in poly‐DOL QSSE (top), while poly‐DOL_0.9_‐TFEP_0.1_ QSSE maintains an amorphous structure (bottom), confirming successful crystallinity suppression through TFEP incorporation. For consistency throughout this study, all poly‐DOL QSSEs were tested within hours of polymerization to ensure non‐crystallized states.

The electrochemical stability windows of poly‐DOL QSSE and poly‐DOL_0.9_‐TFEP_0.1_ QSSE were evaluated using linear sweep voltammetry (LSV) (Figure 2g). Previous investigations have indicated an oxidation potential of ≈4.1 V (vs. Li^+^/Li) for liquid electrolytes,^[^ ^8b,d^ ^]^ which serves as a benchmark for response current. The LSV analysis (Figure 2g) demonstrated a significant enhancement in the oxidation potential of poly‐DOL_0.9_‐TFEP_0.1_ QSSE, reaching 4.6 V. This increase in electrochemical stability was further supported by DFT calculations (Figure 2h). These computational results showed a lower highest occupied molecular orbital (HOMO) energy for the poly‐DOL_0.9_‐TFEP_0.1_ backbone (−6.88 eV) compared to DOL (−6.71 eV), suggesting a potentially greater compatibility with high‐voltage cathode materials for poly‐DOL‐based QSSEs compared to their DOL counterparts.

### 2.3 Analysis of Protective Effects of LiNO_3_‐Containing Poly‐DOL‐Based QSSE on LMA

**Figure 3:**
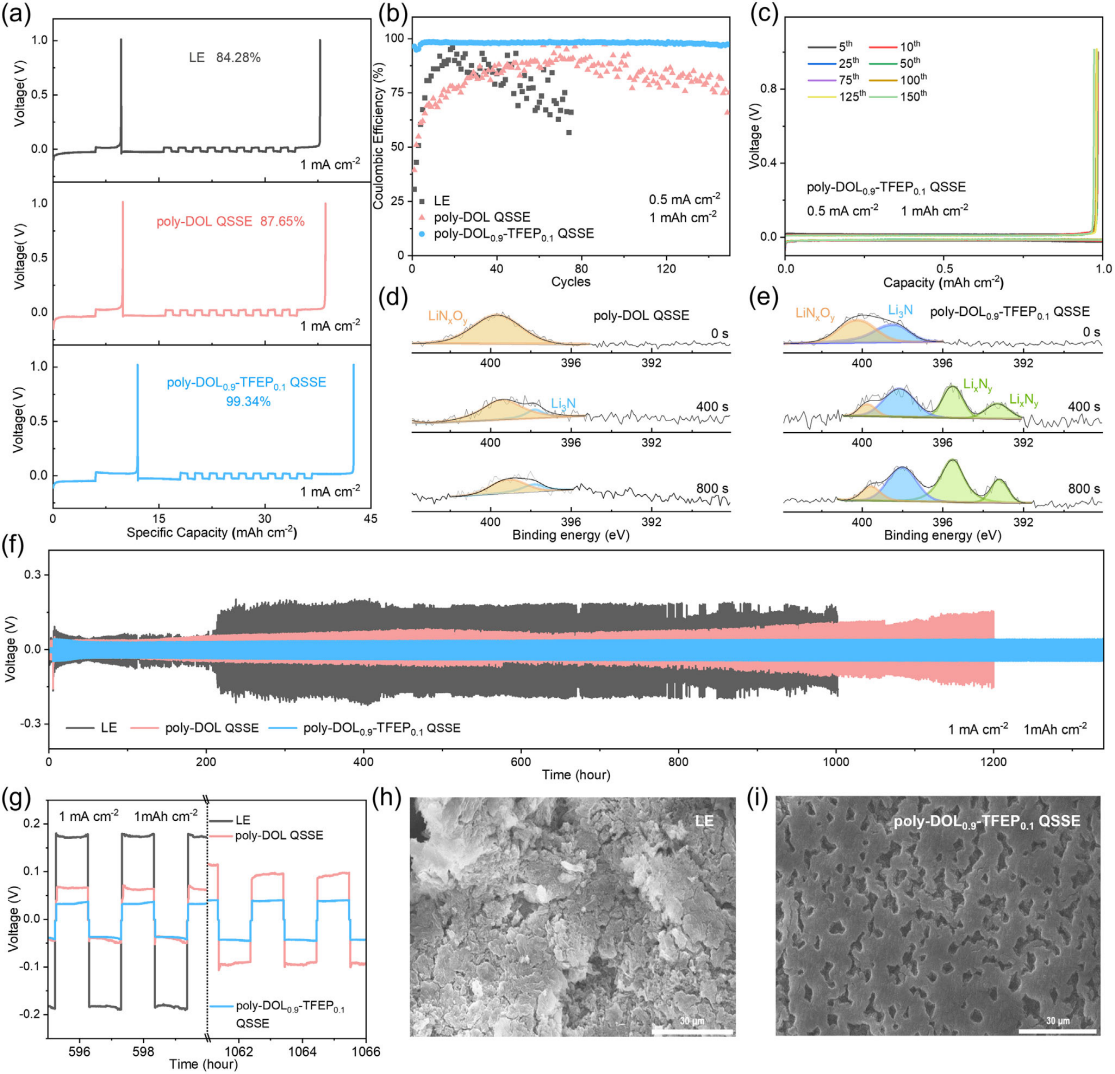
Electrochemical performance and interfacial analysis of LMAs in various electrolyte systems. a) The voltage responses of Li|Cu cells show distinct behaviors in three configurations: LE (top), poly‐DOL QSSE (middle), and poly‐DOL_0.9_‐TFEP_0.1_ QSSE (bottom). Extended cycling stability at 0.5 mA cm^−2^ and 1 mAh cm^−2^ is shown in b) for Li|Cu cells, while c) provides detailed charge–discharge characteristics of the Li|poly‐DOL_0.9_‐TFEP_0.1_ QSSE|Cu cell. Surface chemistry evolution is demonstrated through N 1s XPS depth profiling of cycled LMA interfaces in d) poly‐DOL QSSE and e) poly‐DOL_0.9_‐TFEP_0.1_ QSSE after 100 cycles in Li|LiFePO_4_ cells. Long‐term stability is evident in (f,g) through voltage profiles of Li|Li symmetrical cells across electrolyte variants. Morphological examination via SEM reveals distinct surface features of cycled LMAs after 100 cycles in Li|LiFePO_4_ cells with h) LE and i) poly‐DOL_0.9_‐TFEP_0.1_ QSSE.

To understand the interfacial stabilization mechanism of poly‐DOL_0.9_‐TFEP_0.1_ QSSE, we compared the CEs of Li|Cu half‐cells using four electrolyte systems: LE, LE‐N (0.2 m LiNO_3_ added to LE), poly‐DOL QSSE, and poly‐DOL_0.9_‐TFEP_0.1_ QSSE (**Figure** **3**a; Figure S18, Supporting Information). In these experiments, 6 mAh cm^−2^ of lithium was deposited onto copper foil and then galvanostatically stripped up to 1.0 V to eliminate the influence of potential contaminants on the copper surface. Subsequently, 10 1‐h cycles were performed at an area‐specific capacity of 1 mAh cm^−2^. The final CE of the poly‐DOL_0.9_‐TFEP_0.1_ QSSE cell was 99.34%, significantly surpassing that of the poly‐DOL QSSE cell (87.65%), with further details provided in the Section S1 (Supporting Information). Long‐term cycling of Li|Cu cells was conducted at a current density of 0.5 mA cm^−2^ and an areal capacity of 1 mAh cm^−2^. As illustrated in Figure 3b, the CE of the LE cell fluctuated and rapidly declined to ≈80% after 31 cycles. The LE‐N cell exhibited CEs above 95% during the initial 30 cycles, followed by a gradual decrease to around 85% after the 40th cycle (Figures S19 and S20, Supporting Information). In contrast, the poly‐DOL_0.9_‐TFEP_0.1_ QSSE cell demonstrated a stable CE with values exceeding 97.40% even after 150 cycles (Figure 3c), suggesting the enhanced synergistic effects of LiNO_3_ and poly‐DOL_0.9_‐TFEP_0.1_ QSSE on improving the long‐term stability of LMA surfaces.

X‐ray photoelectron spectroscopy (XPS) was used to characterize the LMAs, comparing the surface chemistry induced by poly‐DOL QSSE and poly‐DOL_0.9_‐TFEP_0.1_ QSSE. The LMA from the poly‐DOL QSSE sample showed minimal nitrogen content (0.4 at.%) after 600 s of etching (Figures S21–S23, Supporting Information). Further analysis of the N 1s spectra (Figure 3d) revealed that the nitrogen‐based compounds likely resulted from LiTFSI reduction,^[^ ^26^ ^]^ which contained predominantly LiN_x_O_y_ (399.9 eV). In contrast, the LMA from the poly‐DOL_0.9_‐TFEP_0.1_ QSSE sample had a higher N content (≈2.2 at.%), which is attributed to the irreversible decomposition of LiNO_3_. This interface was primarily composed of LiN_x_O_y_ and Li_3_N (398.2 eV), with the latter, along with Li_x_N_y_ species (395.5 eV, 393.3 eV), becoming more prominent with etching depth (Figure 3e). These nitrogen‐based compounds, particularly Li_3_N and Li_x_N_y_, are known to inhibit parasitic reactions and dendrite formation on LMAs, thereby enhancing the CE and overall battery stability.^[^ ^10^, ^27^ ^]^

To evaluate the protective effect of poly‐DOL_0.9_‐TFEP_0.1_ QSSE on the LMA, we cycled symmetric Li|electrolyte|Li cells at a current density of 1 mA cm^−2^ and a capacity of 1 mAh cm^−2^ comparing three electrolytes, namely the LE, poly‐DOL QSSE, and poly‐DOL_0.9_‐TFEP_0.1_ QSSE. As shown in Figure 3f, all cell configurations exhibited relatively stable overpotentials during the first 200 h of cycling. However, the LE cell later displayed a sharp increase in overpotential, exceeding 170 mV. In contrast, the poly‐DOL QSSE cell demonstrated improved stability, maintaining an overpotential of ≈60 mV for an extended period, with a notable increase occurring only after 800 h. This observation underscores the beneficial impact of the QSSE on LMA stabilization. Remarkably, the poly‐DOL_0.9_‐TFEP_0.1_ QSSE cell consistently maintained an overpotential below 47 mV, even after 1,300 h of cycling (Figure 3g). This superior performance suggests a synergistic effect between the LiNO_3_‐derived nitrogen‐rich SEI and poly‐DOL‐based QSSE in stabilizing the LMA. Morphological analysis of the LMA surfaces revealed significant differences across the electrolyte systems (Figure 3h,i; Figure S24, Supporting Information). Specifically, the LMA surface in the poly‐DOL_0.9_‐TFEP_0.1_ QSSE cell was smoother (Figure 3i) compared to those of the LE and poly‐DOL QSSE cells.

### 2.4 Performance of LiNO_3_‐Containing QSSE in Coin‐Type Cells and Pouch‐Type Cells

**Figure 4:**
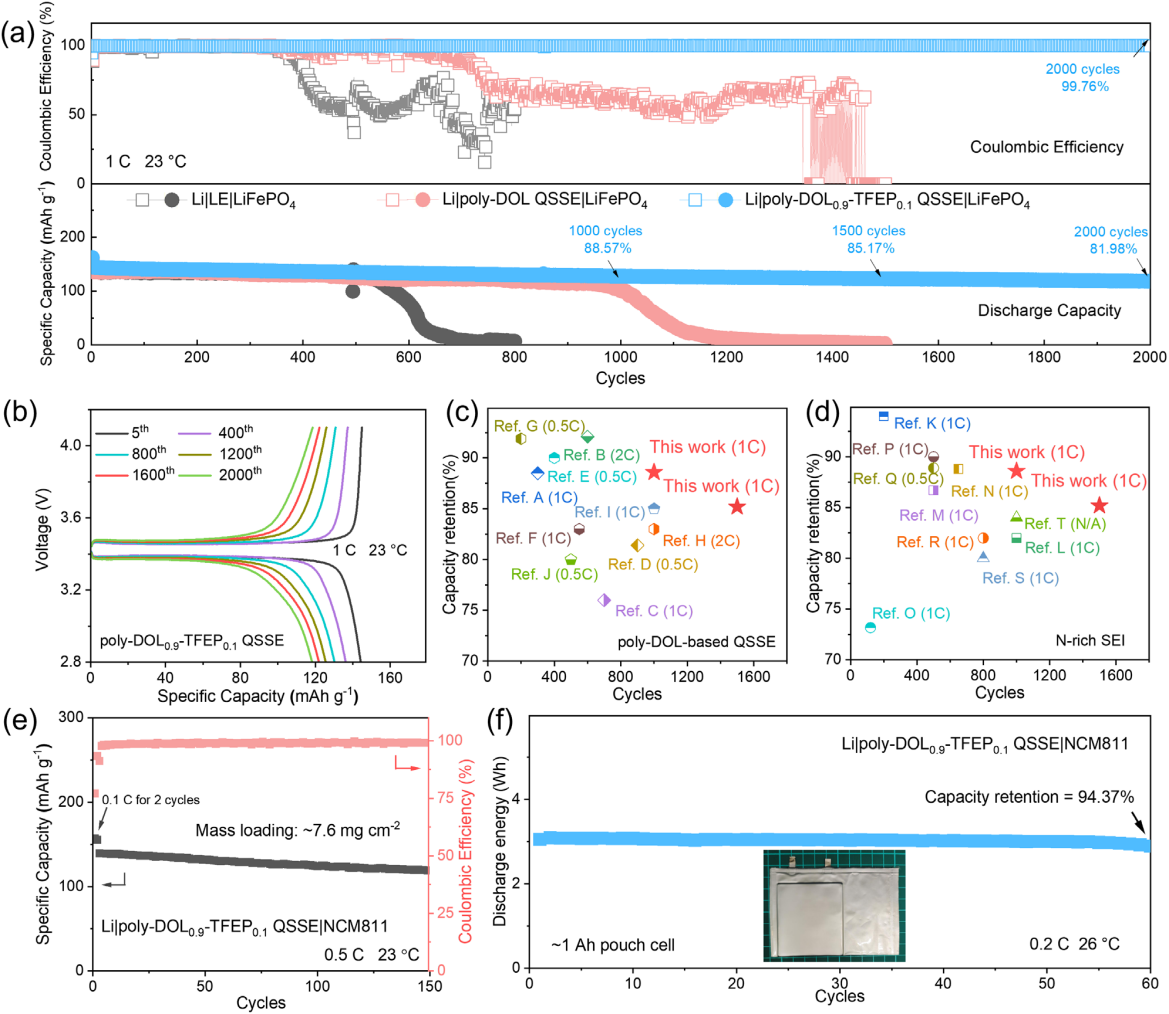
Electrochemical performance. a) Long‐term cycling stability of Li|LiFePO_4_ coin cells at 1C using three electrolyte systems: LE, poly‐DOL QSSE, and poly‐DOL_0.9_‐TFEP_0.1_ QSSE. b) Voltage profiles during charge–discharge cycles of the Li|poly‐DOL_0.9_‐TFEP_0.1_ QSSE|LiFePO_4_ cell. Performance benchmarking against reported Li|LiFePO_4_ cells comparing capacity retention, cycle life, and rate capabilities for systems utilizing c) poly‐DOL‐based QSSE^[^ ^8^, ^13^, ^14^, ^28^ ^]^ and d) N‐rich SEI layer.^[^ ^10^, ^29^ ^]^ Scale‐up validation through e) cycling performance of Li|NCM811 cells with high mass loading (7.6 mg cm^−2^) using poly‐DOL_0.9_‐TFEP_0.1_ QSSE at 0.5C, and f) extended cycling of Li|NCM811 pouch cells at 0.1C charge/0.2C discharge rates after initial formation at 0.05C.

To investigate the impact of electrolyte composition on cell performance, we assembled coin‐type batteries and conducted extensive cycling tests. For QSSE‐based batteries, the liquid‐state precursor solution was initially introduced into the cells to ensure thorough infiltration before in situ polymerization (Figure S25, Supporting Information). We evaluated the performance of Li|LiFePO_4_ cells cycled at 1C (170 mA g^−1^) using three separate electrolyte systems: i) LE, ii) poly‐DOL QSSE, and iii) poly‐DOL_0.9_‐TFEP_0.1_ QSSE. **Figures** **4**a and S26 (Supporting Information) show the cycling performance of these cells. The LE cell initially delivered a promising specific capacity of 136.3 mAh g^−1^ (3rd cycle). However, both its capacity and CE declined quickly after 500 cycles, reaching 77.7 mAh g^−1^ and 59.1%, respectively, by the 600th cycle. The poly‐DOL QSSE cell exhibited a comparable initial capacity but showed improved stability over 800 cycles, at which point the CE dropped below 65%. The poly‐DOL_0.9_‐TFEP_0.1_ QSSE cell demonstrated significantly better performance, including an initial specific capacity of 144.3 mAh g^−1^ (3rd cycle) at 1 C (Figure 4b) and strong capacity retention, maintaining 88.6% and 85.2% of its initial capacity at the 1000^th^ and 1500th cycles, respectively. This capacity retention outperforms most previously reported poly‐DOL‐based QSSE cells or cells with modified nitrogen‐rich SEIs (Figure 4c,d). Furthermore, the CE of this LiNO_3_‐containing QSSE cell remained consistently above 99.7%, even after 2,000 cycles. To demonstrate the broader applicability of this LiNO_3_‐containing QSSE, we fabricated batteries with high‐loading cathodes, such as LiFePO_4_ (mass loading of 10.2 mg cm^−2^) and NCM811 (mass loading of 7.6 mg cm^−2^). During full‐cell cycling, the poly‐DOL_0.9_‐TFEP_0.1_ QSSE demonstrated limited cycling stability when paired with high‐voltage cathodes (above 4.2 V vs. Li/Li^+^). This limitation can be attributed to the inherently low oxidative stability of residual liquid DOL within the poly‐DOL_0.9_‐TFEP_0.1_ QSSE (Table S4, Supporting Information). Consequently, we set a charge cut‐off voltage of 4.2 V (vs. Li/Li⁺) for the NCM811 coin cells. As illustrated in Figures S27 and S28 (Supporting Information), and Figure 4e, these cells exhibited robust cycling stability.

To evaluate the practical viability of poly‐DOL_0.9_‐TFEP_0.1_ QSSE, we fabricated and tested a pouch‐type Li|NCM811 cell with an approximate capacity of 1 Ah (Figure 4f; Figure S31, Supporting Information). The cathode had an active material loading of 11.27 mg cm^−2^ per side, nearing commercial standards. This cell achieved a specific energy of 302 Wh kg^−1^ (Table S7, Supporting Information) and maintained capacity retention of 94.37% after 60 cycles at 0.2C, relative to the second cycle. These results demonstrate the significant potential of poly‐DOL_0.9_‐TFEP_0.1_ QSSE for practical lithium metal batteries, combining high energy density with impressive cycling stability in a commercially relevant format.

LiNO_3_ is also a recognized additive for Li–S batteries, effectively mitigating the “shuttle effect” caused by parasitic reactions between soluble polysulfides and the LMA. To evaluate the efficacy of the LiNO_3_‐containing QSSE, Li–S cells were constructed using both poly‐DOL QSSE and poly‐DOL_0.9_‐TFEP_0.1_ QSSE (Figure S32, Supporting Information). The Li–S battery using poly‐DOL_0.9_‐TFEP_0.1_ QSSE demonstrated significantly higher capacity and a superior average CE of 97.16%, compared to the poly‐DOL QSSE, which exhibited an average CE of only 68.23%.

These results collectively highlight the superior performance and versatility of the LiNO_3_‐containing, poly‐DOL_0.9_‐TFEP_0.1_ QSSE system, demonstrating its potential to significantly advance the development of high‐performance, long‐lasting lithium metal batteries.

## 3 Conclusion

This study presents a transformative strategy that harnesses polymerization enthalpy to overcome fundamental limitations in poly‐DOL‐based QSSEs. Through strategic co‐polymerization with high‐polymerization‐enthalpy TFEP, we successfully resolved the inherent incompatibility between LiNO_3_ and poly‐DOL‐based QSSEs while simultaneously suppressing polymer crystallization. The synergistic protection mechanism, which combines LiNO_3_‐derived SEI with poly‐DOL‐based QSSE, achieves notable performance in both coin and pouch cell formats. The exceptional cycling stability in Li|LiFePO_4_ coin cells exceeds 2,000 cycles with superior capacity retention. An Ah‐scale pouch cell achieved a specific energy of 302 Wh kg^−1^ and a capacity retention of 94.4% over 60 cycles, validating the viability of this approach. Beyond these performance results, our strategy sets a new paradigm for the development of in situ polymerized QSSEs, extending beyond conventional poly‐DOL systems. By introducing polymerization enthalpy as a key design parameter, we demonstrate how molecular‐level engineering can effectively address material incompatibilities while maintaining practical processing. This comprehensive approach not only tackles existing technical challenges but also provides a versatile framework for advancing next‐generation energy storage technologies.

## Conflict of Interest

The authors declare no conflict of interest.

## Supporting information



Supporting Information

## Data Availability

The data that support the findings of this study are available from the corresponding author upon reasonable request.
