# Supplementary material for: Enthalpy‐Driven Molecular Engineering Enables High‐Performance Quasi‐Solid‐State Electrolytes for Long Life Lithium Metal Batteries
Source: Adv Mater. 2025 Apr 7;37(24):2419335. doi: 10.1002/adma.202419335 (PMC12177861; doi:10.1002/adma.202419335)
Supplement: Supplementary file 1 — Supporting Information [file ADMA-37-2419335-s001.docx]

Enthalpy-Driven Molecular Engineering Enables High-Performance Quasi-solid-state Electrolytes for Long Life Lithium Metal Batteries

Zilong Wang,^a^ Longyun Shen,^b^ Yilin Ma,^a^ Ho Mei Law,^c,d^ Shengjun Xu, ^c,d^ Yixin Bi,^a^ Matthew J. Robson,^a^ Yuhao Wang,^a^ André Gröschel,^d,e^ Qing Chen,^a,^* Francesco Ciucci^a,c,d,^*

1. Department of Mechanical and Aerospace Engineering, The Hong Kong University of Science and Technology, Clear Water Bay, Kowloon, Hong Kong S.A.R., China
2. Division of Emerging Interdisciplinary Areas, The Hong Kong University of Science and Technology, Clear Water Bay, Kowloon, Hong Kong S.A.R., China
3. University of Bayreuth, Chair of Electrode Design for Electrochemical Energy Systems, Weiherstraße 26, 95448 Bayreuth, Bayreuth, Germany
4. University of Bayreuth, Bavarian Center for Battery Technology (BayBatt), Universitätsstraße 30, 95447 Bayreuth, Germany
5. Polymer Materials for Energy Storage (PES) and Macromolecular Chemistry, University of Bayreuth, Weiherstraße 26, 95448 Bayreuth, Germany

* Corresponding author e-mail:

francesco.ciucci@ust.hk or francesco.ciucci@uni-bayreuth.de (Francesco CIUCCI).

* Corresponding author e-mail:

chenqing@ust.hk (Qing CHEN).

**Methods**

Experiments

*Materials*: 1,1,1-Trifluoro-2,3-epoxypropane (TFEP, 98%), propylene oxide (PO, 99%), and 1,2-butylene oxide (BO, 99%) were purchased from Aladdin and dried over 4 Å molecular sieves prior to use. 1,3-Dioxolane (DOL) was purchased from Canrd Technology Co. Ltd., distilled at 85°C to eliminate polymerization inhibitors, and subsequently dried over 4 Å molecular sieves. Lithium bis(trifluoromethanesulfonyl)imide (LiTFSI, 99.95%), lithium hexafluorophosphate (LiPF_6_, 99%), lithium nitrate (LiNO_3_, 99.99%), and tin(II) fluoride (SnF_2_, 99%) were sourced from Sigma-Aldrich and used as received.

*Preparation of QSSEs*: LiTFSI (1 M) and LiNO_3_ (0.2 M) were initially dissolved in DOL. The mixture was then stirred for 30 minutes at room temperature. Subsequently, a three-membered cyclic ether (PO, BO, or TFEP) was added to the mixture. After another 30 minutes of stirring at room temperature, initiators (0.5 wt% SnF_2_ and 2.5 wt% LiPF_6_) were introduced. The resulting solution was injected into either a coin-type (55 µL) or pouch-type cell (2.2 g), which was then sealed. The precursor solution underwent spontaneous *in situ* polymerization to form the QSSEs. The electrolyte preparation and the coin-type cell fabrication were conducted within a glove box (Mikrouna, O_2_ < 0.01 ppm, H_2_O < 0.01 ppm) under an ultrapure Ar atmosphere (≥99.999%, Air Products).

*Li-Cu & Li-Li batteries:* To evaluate the CEs in relation to the LMA, Li|Cu half-cells were assembled with Cu serving as the working electrode. A pre-conditioning cycle was conducted by electroplating a predetermined quantity of lithium (Q_T_) onto the Cu surface (Q_T_ = 6 mAh cm^-2^ at 1 mA cm^-2^); this step was followed by a complete stripping process to 1.0 V (*vs.* Li/Li^+^). This procedure aimed to eliminate contaminants from the Cu surface. Subsequently, Li was replated onto the Cu electrode (Q_T_ = 6 mAh cm^-2^ at a current density of 1 mA cm^-2^), followed by 10 cycles of stripping and re-plating at specified amounts of lithium (Q_C_= 1 mAh at a current density of 1 mA cm^-2^). Finally, a final stripping step to 1.0 V was performed to remove any remaining lithium (Q_S_) from the Cu electrode. The CE was calculated using the following formula:

| $CE=\frac{10\cdot Q_{C}+Q_{S}}{10\cdot Q_{C}+Q_{T}}$ | (1) |
| --- | --- |

For long-term CEs evaluation, Li|Cu cells were charged and discharged at current densities of 0.5 mA cm^-2^ and areal capacity of 1 mAh cm^-2^.

Li|Li symmetrical cells were assembled with Li foil serving as working and counter electrodes. Initially, the cells were subjected to galvanostatic charge-discharge cycles at a current density of 0.2 mA cm^-2^ and a capacity of 0.2 mAh cm^-2^ for two cycles. Subsequent long-term cycling tests were conducted at increased current densities and capacities of 1 mA cm^-2^ and 1 mAh cm^-2^, respectively.

*Preparation of LiFePO_4_ & NCM811 cathode for coin-type batteries*: The LiFePO_4_ slurry was prepared by blending LiFePO_4_ powder (Canrd Technology Co. Ltd.), conductive carbon black (TIMCAL Super P, MTI), and polyvinylidene difluoride (PVDF) binder at a weight ratio of 8:1:1 in N-methyl-2-pyrrolidone (NMP, 99.9%, MTI) solvent. Subsequently, the slurry was stirred magnetically for 7 hours before being cast onto aluminum foil. Following drying at 90°C for 12 hours, the resulting LiFePO_4_-based cathode layer was shaped into disks (12 mm in diameter) with a mass loading ranging from 1.1 to 1.5 mg cm^−2^ (LiFePO_4_). High mass loading cathodes, comprising either LiFePO_4_ (10.2 mg cm^−2^) or NMC811 (7.6 mg cm^−2^), were also prepared by blending active material/Super P/PVDF in a weight ratio of 9:0.5:0.5.

*Electrochemical characterizations:*

To measure the ionic conductivity (σ) of QSSEs, the precursor solution was infiltrated into a glass fiber separator which was sandwiched between stainless steel (SS) disks to form a SS|QSSE|SS symmetric cell. The ionic conductivity was calculated using the formula:

| $\sigma=\frac{L}{RA}$ | (2) |
| --- | --- |

where A is the cross-sectional area of the stainless steel (SS) electrodes, L is the thickness of glass fiber, and R is the ohmic resistance of the SS|QSSE|SS symmetric cell, measured using electrochemical impedance spectroscopy (EIS). EIS measurements were conducted within the frequency range of 1 Hz to 7 MHz with an AC amplitude of 10 mV and 6 points per decade utilizing a Bio-Logic VSP-300 electrochemical workstation. The transference number of lithium ions (t_Li+_) was calculated based on chronoamperometric and EIS characterization of Li|Li symmetrical cells, using the following formula^[1]^:

| $t_{\mathrm{Li}^{+}}=\frac{I_{ss}(\Delta V-I_{0}R_{0})}{I_{0}(\Delta V-I_{ss}R_{ss})}$ | (3) |
| --- | --- |

where I_0_ and I_ss_ represent the initial and steady-state currents, respectively, acquired by chronoamperometry and Δ𝑉 denotes the polarization potential of 10 mV. Furthermore, R_0_ and R_ss_​ are the initial and steady-state resistances, respectively, as measured using EIS. To determine the oxidation potential, linear sweep voltammetry (LSV) was performed on a Li|QSSE|SS cell within a potential range of 1-6 V *vs.* Li^+^/Li and a scan rate of 5 mV s^−1^. Both the ionic conductivity and LSV tests were conducted utilizing a Bio-Logic VSP-300 electrochemical workstation. For the cycling performance of the batteries, a CT2001A (LANHE) battery cycler was used under controlled temperature conditions.

*Physical characterizations:* Fourier-transform infrared spectroscopy (FTIR) data were obtained using a Bruker Vertex 70 FTIR spectrometer. To analyze the ^1^H and ^19^F spectra, nuclear magnetic resonance (NMR) spectroscopy was conducted using a Bruker 600MHz instrument. Scanning electron microscopy (SEM) analysis of the surface morphology of LMA was performed using a JEOL-6390 instrument. X-ray photoelectron spectroscopy (XPS) measurements were taken with a PHI5600 X-ray photoelectron spectrometer. The cycled Li metal anodes intended for XPS analysis were washed with pure DOL and stored in a glovebox before testing. X-ray diffraction (XRD) measurements were collected over the 2θ range of 5° to 90° using a PANalytical Empyrean diffractometer equipped with Cu K_α_ radiation. Gel permeation chromatography (GPC) measurements were conducted using an Agilent PL-GPC220. Thermo-gravimetric analysis (TGA) measurements were performed using a Q5000-TA from room temperature to 500°C at a heating rate of 10°C min^−1^ in N_2_ (99.996%). Raman tests were conducted using a Raman spectrometer (InVia. Renishaw) at an excitation wavelength of 785 nm. Rheometric experiments were carried out using a parallel-plate geometry with the strain-controlled rheometer Waters DHR-2.

Calculations

*MD calculations*: MD simulations were conducted using GROMACS 2021.6^[2]^ with the OPLS-AA force field^[3]^, and RESP charges derived from Multiwfn^[4]^. Initially, the simulation box was set to 120×120×120 Å, with initial atomic coordinates generated using Packmol^[5]^. The temperature during NPT equilibration and NVT trajectory production was maintained thorough Nosé-Hoover temperature coupling. The pressure during NPT equilibration was regulated at 1 bar using Parrinello-Rahman pressure coupling^[6]^. Electrostatic interactions were handled with particle-mesh Ewald algorithm using a 1.0 nm cut-off^[7]^. Firstly, two NPT relaxation steps were set up at 450 K for 1 ns and then at 298 K for 1 ns to allow the electrolyte systems to reach equilibrium. This was followed by a 5.0 ns NVT relaxation at 298 K, and then a 5.0 ns NPT equilibration step. After these steps, a production simulation of 20 ns was conducted, all using a 1 fs timestep.

*DFT calculations:* Polymerization enthalpy calculations were performed using Gaussian 16^[8]^ software. The geometry optimizations incorporated Becke’s three-parameter exchange functional in conjunction with the Lee-Yang-Parr nonlocal correlation functional (B3LYP)^[9]^, utilizing a 6-311G(d,p) basis set^[10]^. Enthalpy computations were performed using the cc-pVTZ basis set^[11]^ to enhance calculation accuracy. Geometry optimization and HOMO/LUMO calculations of poly-DOL and poly-DOL_0.9_-TFEP_0.1_ were performed with ORCA^[12]^ (B3LYP^[9]^-D3BJ^[13]^/def2-TZVP^[14]^) and visualized using Avogadro^[15]^.

**Figures & tables.**


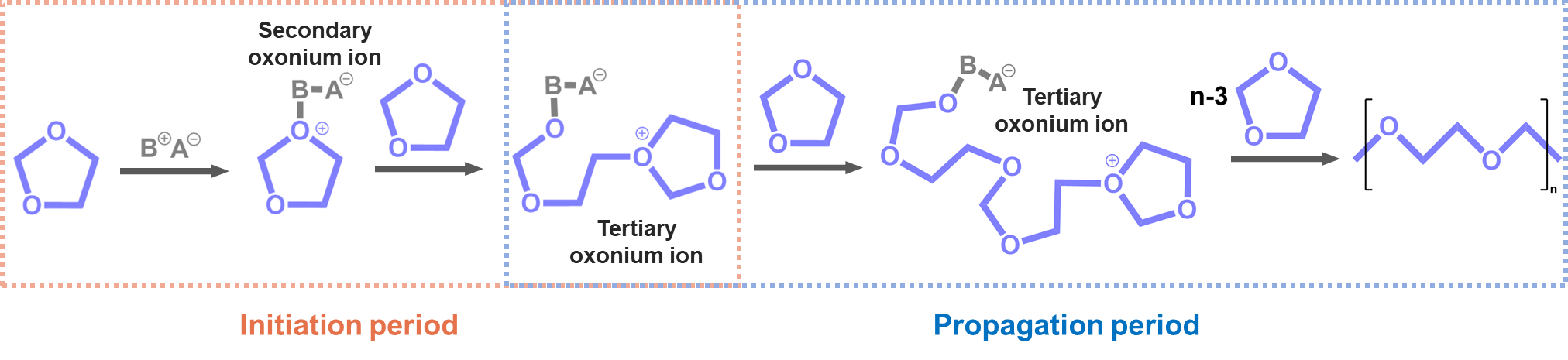
 Fig. S1 Schematics illustrating the ROP of poly-DOL.

***Ring-opening polymerization (ROP) process of 1,3-dioxolane (DOL)***

The ROP of DOL, similar to other cyclic ethers, proceeds through distinct initiation and propagation phases. The process begins with the interaction between a Lewis acid and the polar oxygen atom of a DOL monomer. This interaction disturbs the electronic configuration of DOL, leading to the formation of an ion pair and the generation of a secondary oxonium ion. The newly formed secondary oxonium ion then reacts with another DOL monomer, resulting in the creation of a tertiary oxonium ion. This tertiary oxonium ion acts as the active intermediate, driving the polymerization forward. As the reaction progresses into the propagation phase, the tertiary oxonium ion continues to interact with additional DOL monomers, maintaining the formation of new tertiary oxonium ions. This chain-growth mechanism continues, with each subsequent reaction extending the polymer chain until the polymerization is complete^[16]^.


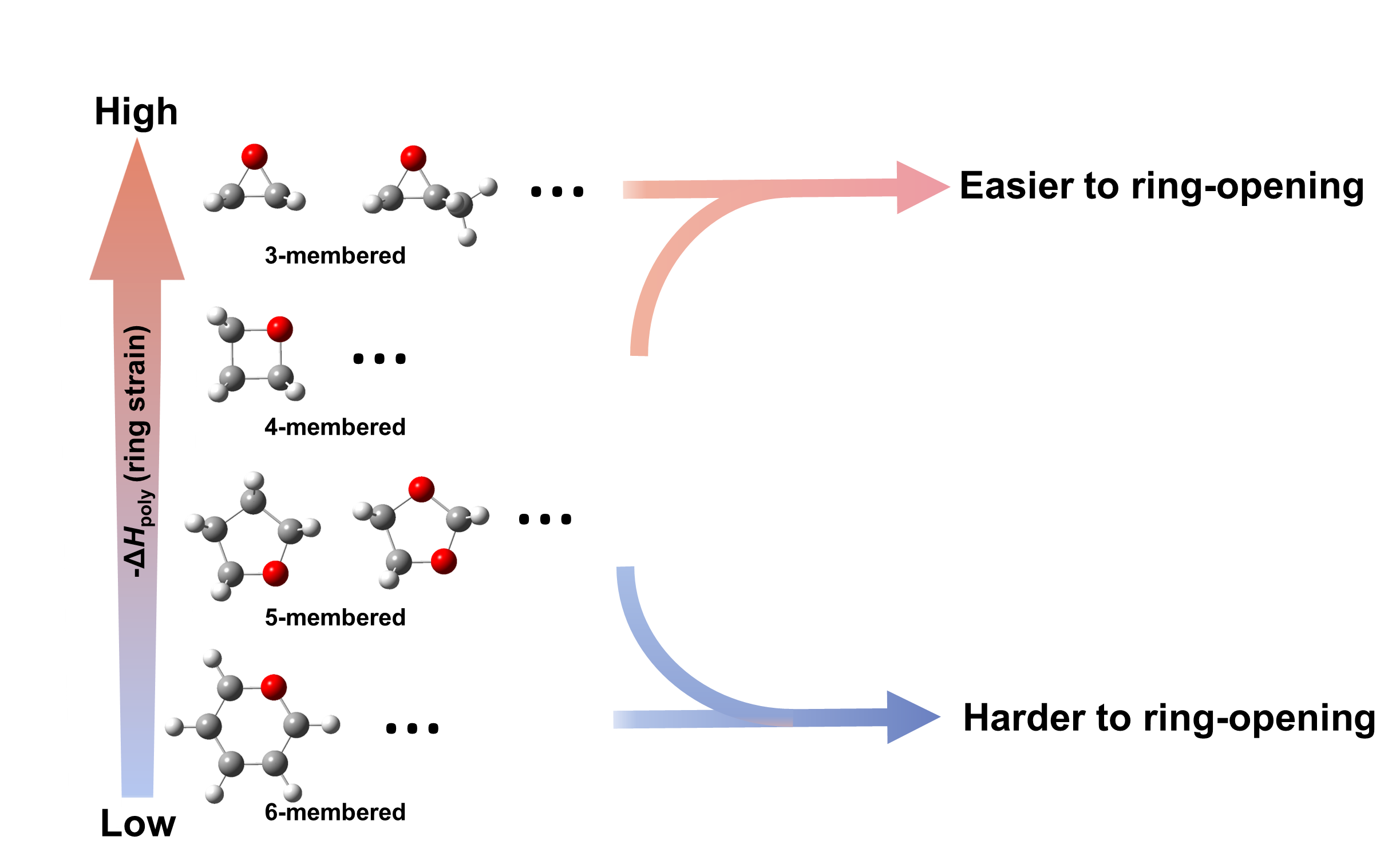


Fig. S2 Schematics illustrating the relationship between ring strain, polymerization enthalpies, and abilities to ROP of 3-, 4-, 5-, and 6-membered cyclic ethers.

***Ring strain and polymerization enthalpy (ΔH_poly_) of cyclic ethers***

The polymerization enthalpy, Δ*H*_poly_, is defined as the difference in energy between the products (polymers) and the reactants (monomers) during the polymerization process. For ROP process of cyclic ethers, Δ*H*_poly_ is intrinsically linked to the ring strain of monomers. Ring strain primarily arises due to deviations between the ideal bond angles required for orbital overlap and the bond angles imposed by the geometry of the ring, which depends on the number of atoms within the ring structure^[16a, 17]^. This ring strain is stored as internal energy within the ring, and during ROP this strain is released as Δ*H*_poly_^[17]^. As shown in Table S1, three-membered and four-membered rings exhibit significant strain, resulting in higher Δ*H*_poly_ values that facilitate the conversion of monomers to polymers, making them easier to undergo ROP. In contrast, five-membered and six-membered rings have lower strain, which leads to smaller Δ*H*_poly_ values and makes them more challenging to polymerize *via* ROP.


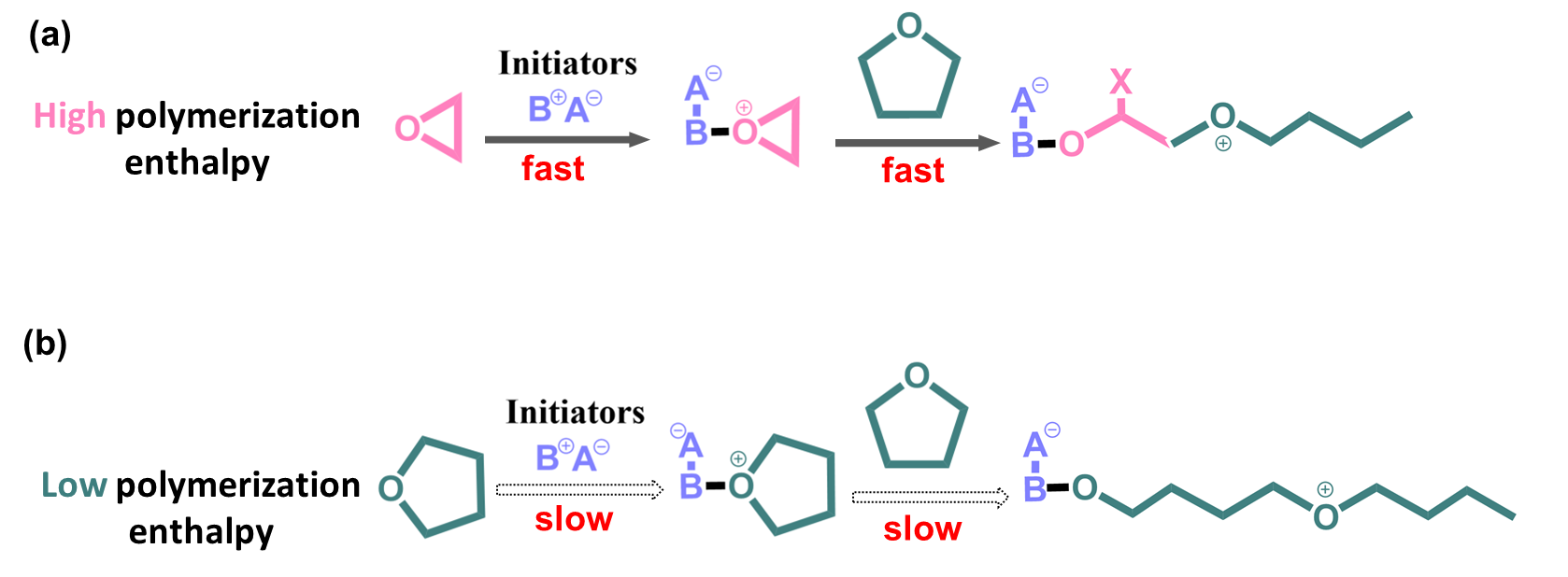


Fig. S3 Schematics illustrating (a) EO was used as a promoter to accelerate the initiation period of THF's ROP process and (b) the basic mechanism of ROP of poly-THF.

***Enthalpy-driven strategy using ethylene oxide (EO) as promoter to accelerate the polymerization of tetrahydrofuran (THF)***

In the synthesis of polytetrahydrofuran (poly-THF), a small amount of EO is typically used as a promoter, a practice rooted in the differing polymerization enthalpies of EO and THF^[16a, 17]^. Both EO and THF, as cyclic ethers, share the same intermediates (tertiary oxonium ions) with DOL during ROP^[16a]^. However, the ring-opening ability of these compounds differ significantly. EO, a three-membered cyclic ether, has a high polymerization enthalpy, which facilitates ring-opening and leads to a quick initiation period in ROP. In contrast, THF, which has a five-membered ring structure, possesses a low polymerization enthalpy, making ring-opening more challenging and resulting in a longer initiation period compared to EO (see Table S1)^[16]^. These unique characteristics allow small amounts of EO to serve effectively as a promoter for THF polymerization. The promotion mechanism involves EO rapidly undergoing ring-opening to generate a tertiary oxonium ion. This intermediate then initiates THF polymerization, significantly shortening the initiation period and speeding up the overall ROP process of THF^[16b, 18]^. In principle, EO could enhance the ROP of LiNO_3_-containing DOL. However, its practical application encounters a major challenge due to its low boiling point of 10.7°C, complicating handling and storage at ambient temperatures.


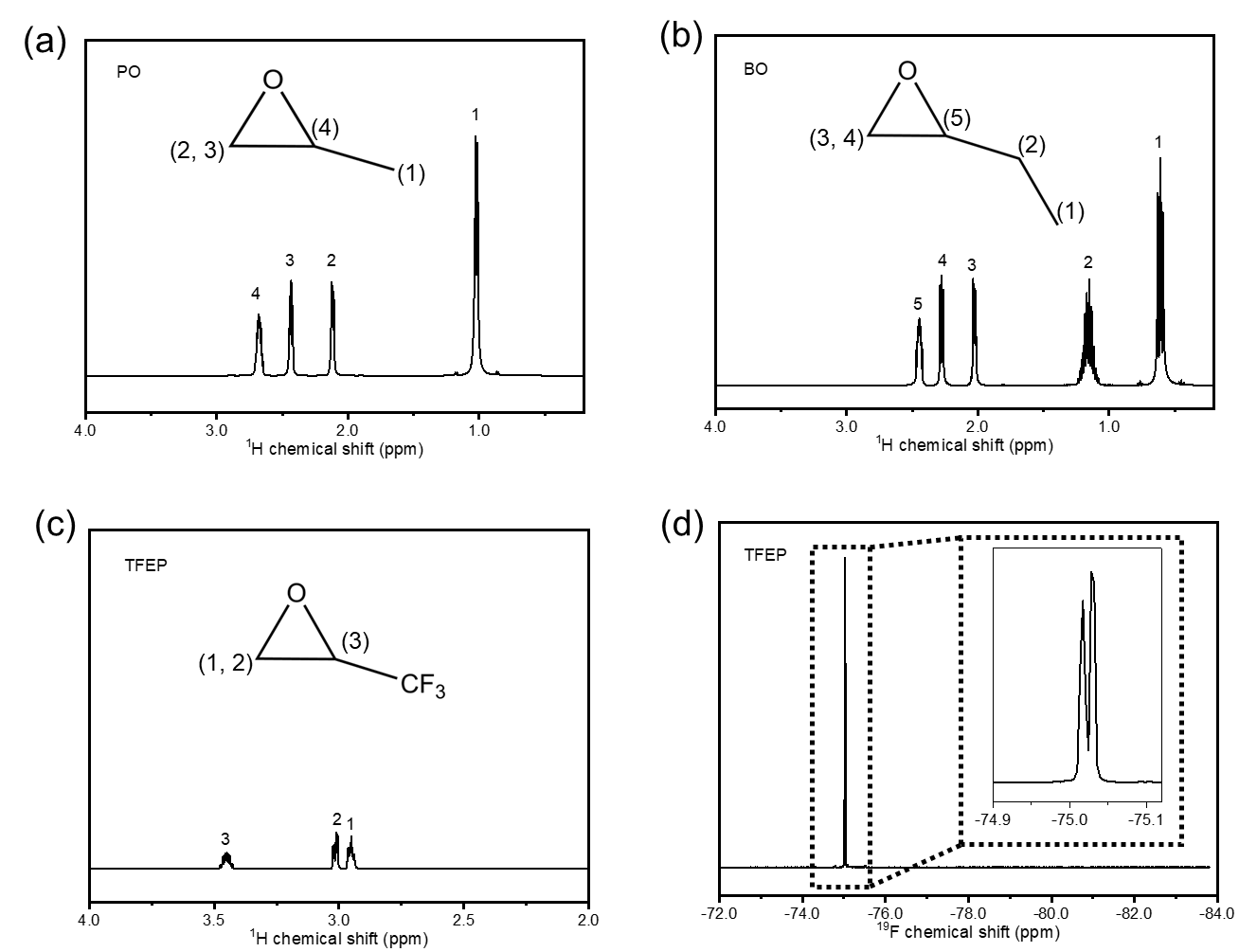


Fig. S4 ^1^H NMR spectrum of (a) propylene oxide (PO), (b) 1,2-butylene oxide (BO), and (c) 1,1,1-trifluoro-2,3-epoxypropane (TFEP). (d) ^19^F NMR spectrum of TFEP.


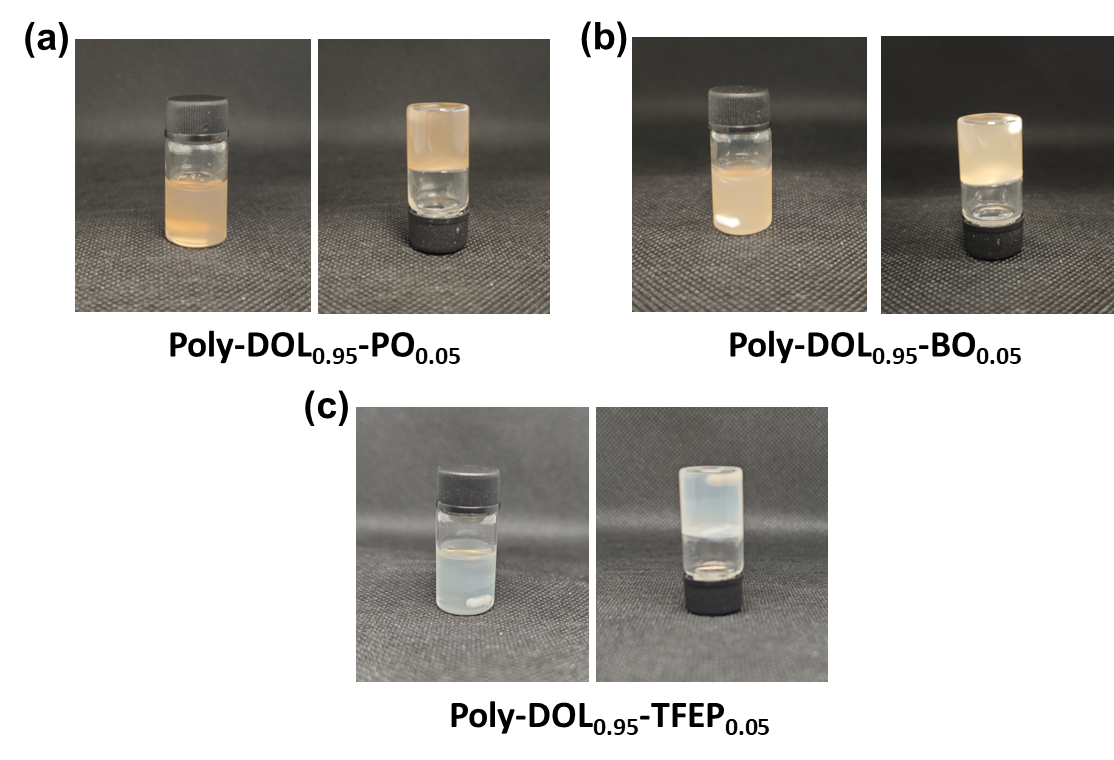


Fig. S5 Digital images of (a) poly-DOL_0.95_-PO_0.05_ QSSE, (b) poly-DOL_0.95_-BO_0.05_ QSSE, (c) poly-DOL_0.95_-TFEP_0.05_ QSSE.

Note: All samples included 1M LiTFSI and 0.2 M LiNO_3_.


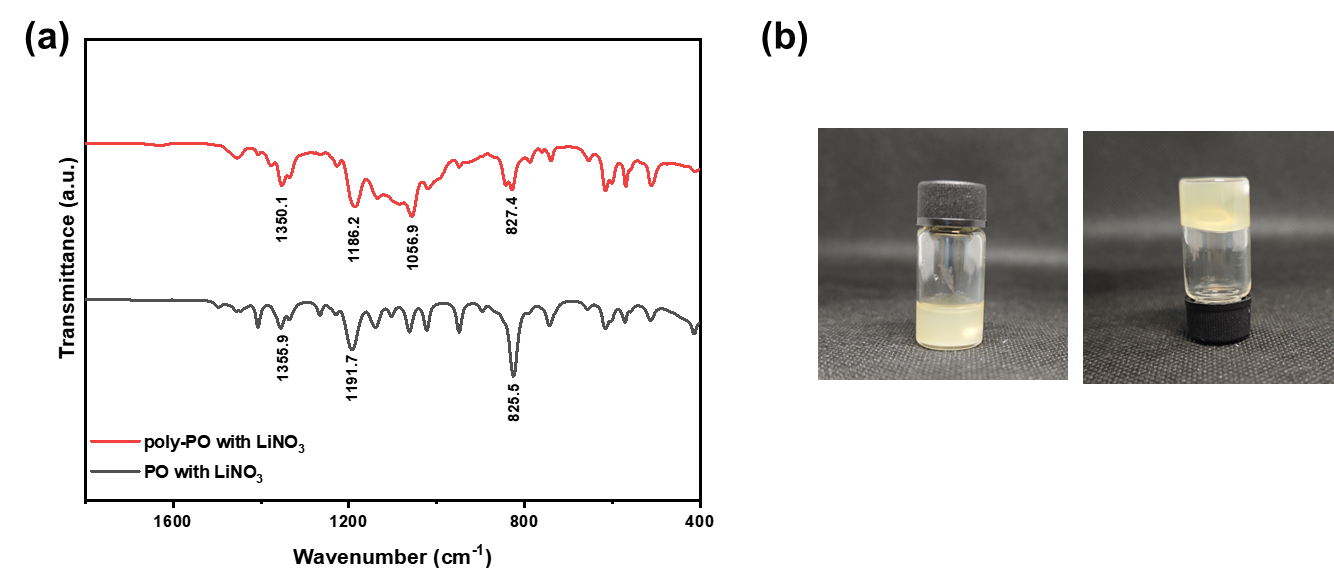


Fig. S6 (a) FTIR spectrum of PO with 0.2 M LiNO_3_ and poly-PO with 0.2 M LiNO_3_. (b) digital images of poly-PO. 1 M LiTFSI was added in this sample.

Note: Both PO and BO (with 1M LiTFSI) have shown polymerization capability in the presence of LiNO_3_, as illustrated in Figures S6 and S7. These findings suggest that both compounds can generate active intermediates, particularly tertiary oxonium ions, when exposed to LiNO_3_. These reactive species are anticipated to assist in the ROP of DOL, potentially alleviating the retarding effect of LiNO_3_ on DOL polymerization. However, the solubility of LiTFSI and LiNO_3_ in pure TFEP presents a significant challenge (Figure S8).


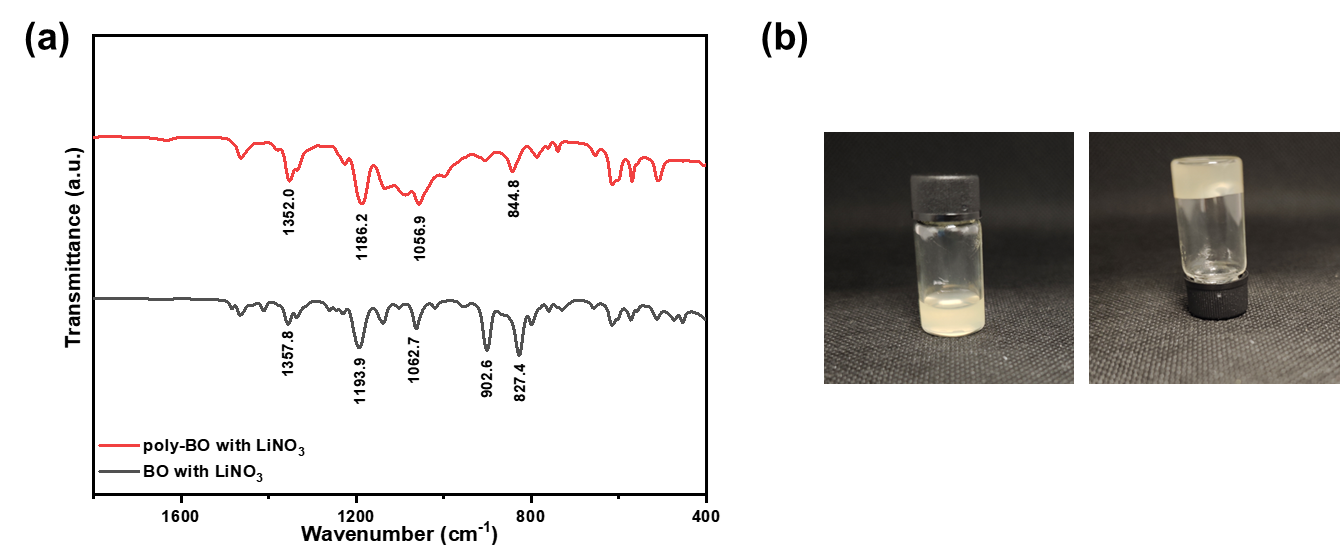


Fig. S7 (a) FTIR spectrum of BO with 0.2 M LiNO_3_ and poly-BO with 0.2 M LiNO_3_. (b) digital images of poly-BO. 1 M LiTFSI was added to this sample.


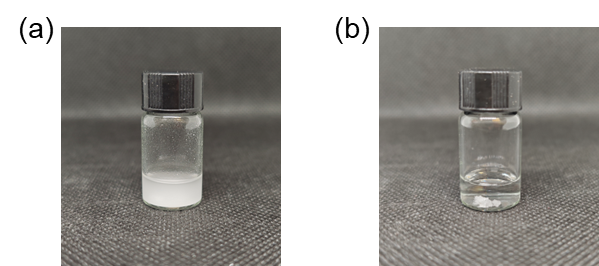


Fig. S8 Digital images of TFEP with (a) 1M LiTFSI and (b) 0.05M LiNO_3_ after 3 days

Fig. S9 FTIR spectrum of TFEP and poly-TFEP.


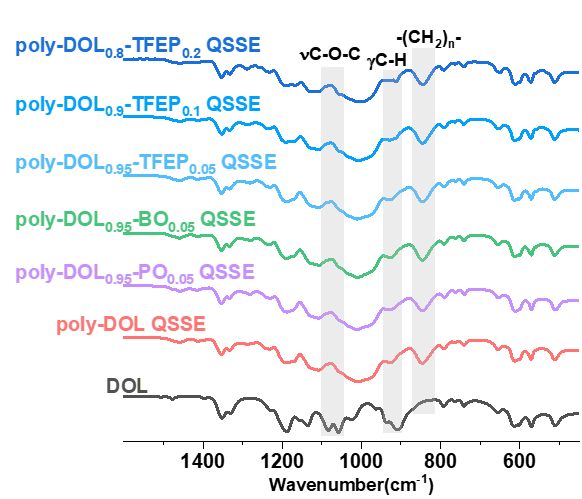


Fig. S10 FTIR spectra of DOL, poly-DOL QSSE, poly-DOL_0.95_-PO_0.05_ QSSE, poly-DOL_0.95_-BO_0.05_ QSSE, poly-DOL_0.95_-TFEP_0.05_ QSSE, poly-DOL_0.9_-TFEP_0.1_ QSSE, and poly-DOL_0.8_-TFEP_0.2_ QSSE. All samples included 1M LiTFSI, while poly-DOL_0.95_-PO_0.05_ QSSE, poly-DOL_0.95_-BO_0.05_ QSSE, poly-DOL_0.95_-TFEP_0.05_ QSSE, poly-DOL_0.9_-TFEP_0.1_ QSSE, and poly-DOL_0.8_-TFEP_0.2_ QSSE also contained 0.2 M LiNO_3_.


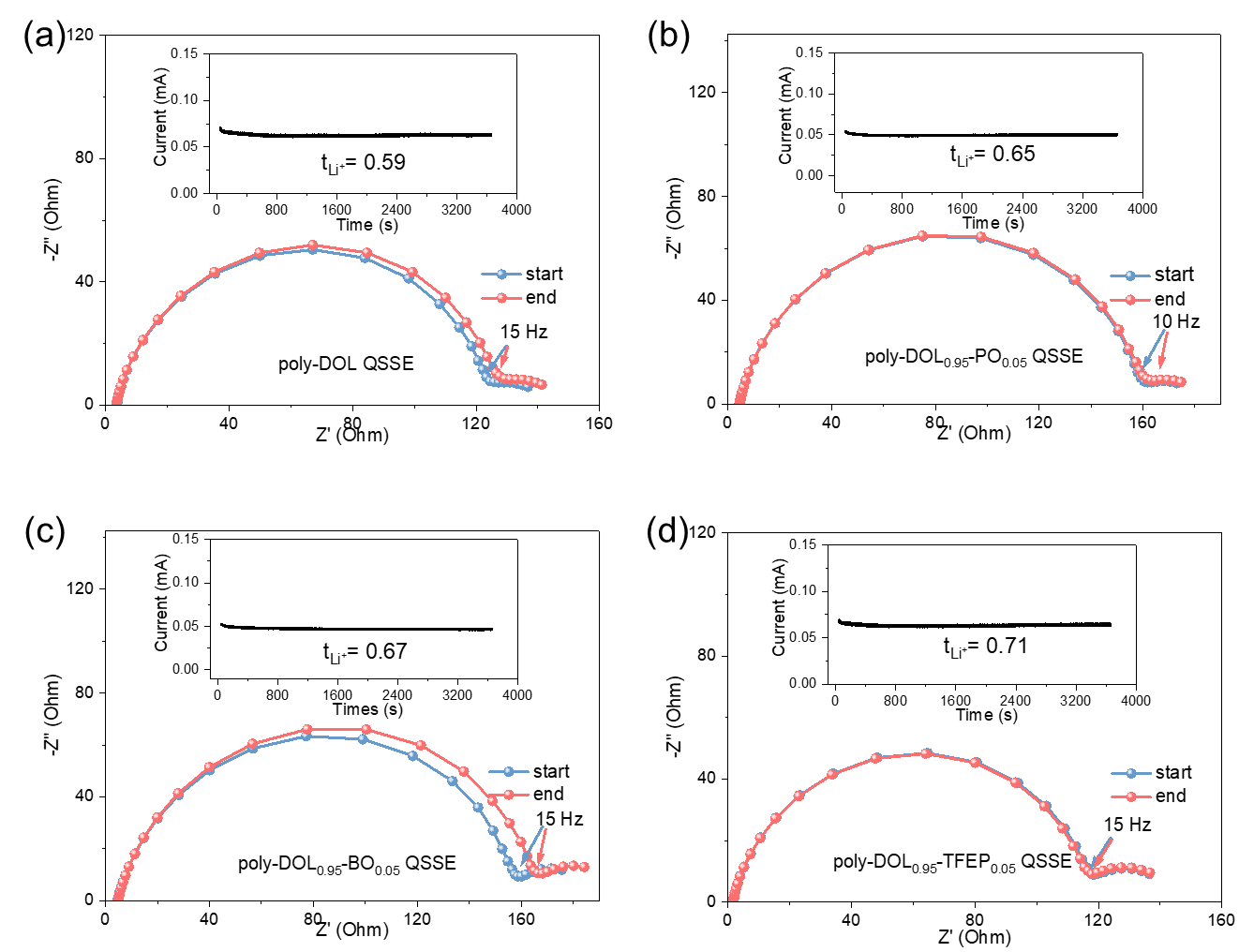


Fig. S11 Nyquist and chronoamperometric plots for (a) poly-DOL QSSE, (b) poly-DOL_0.95_-PO_0.05_ QSSE, (c) poly-DOL_0.95_-BO_0.05_ QSSE, and poly-DOL_0.95_-TFEP_0.05_ QSSE with Li|electrolytes|Li cells.


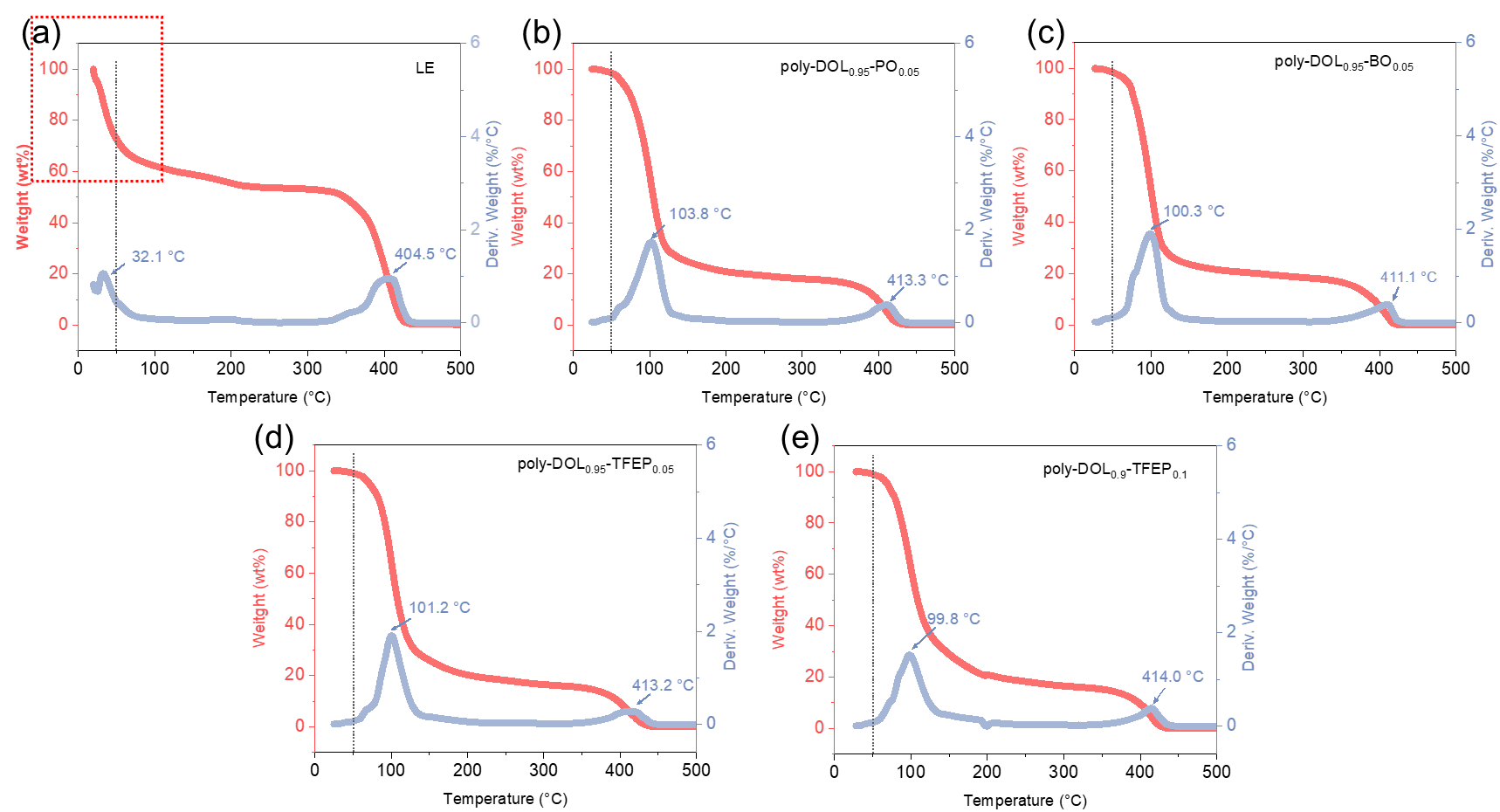


Fig. S12 TGA analysis of various electrolytes. (a) LE; (b) poly-DOL_0.95_-PO_0.05_ QSSE; (c) poly-DOL_0.95_-BO_0.05_ QSSE; (d) poly-DOL_0.95_-TFEP_0.05_ QSSE; (e) poly-DOL_0.9_-TFEP_0.1_ QSSE. Samples (b) to (e) were mixed with 1 M LiTFSI and 0.2 M LiNO_3_.

Note: As highlighted by the red dashed box in Figure S12(a), the LE sample exhibits significant weight loss in the 0–50°C range. In contrast, the QSSE samples (Figures S12(b)-(e)) show substantially lower weight loss, indicating a marked improvement in thermal stability compared to the LE sample. The decomposition of LiTFSI occurs at approximately 400–410 °C.


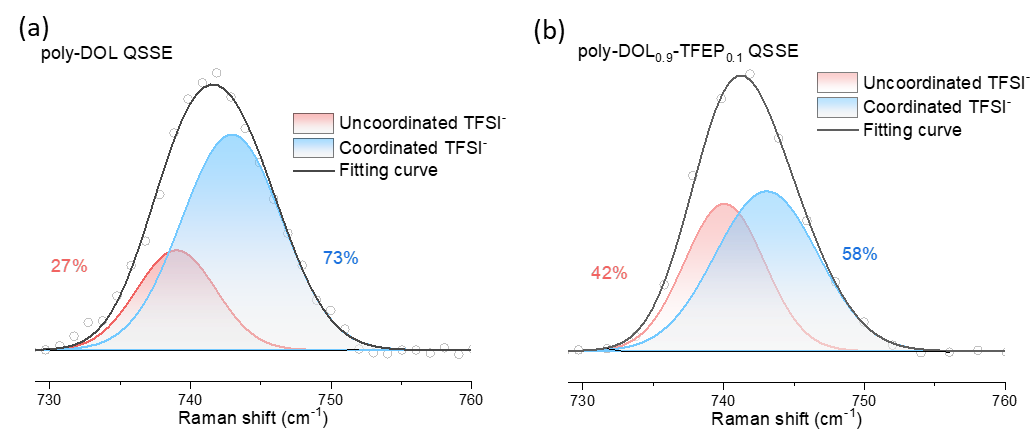


Fig. S13 Raman spectra of poly-DOL QSSE and poly-DOL_0.9_-TFEP_0.1_ QSSE.


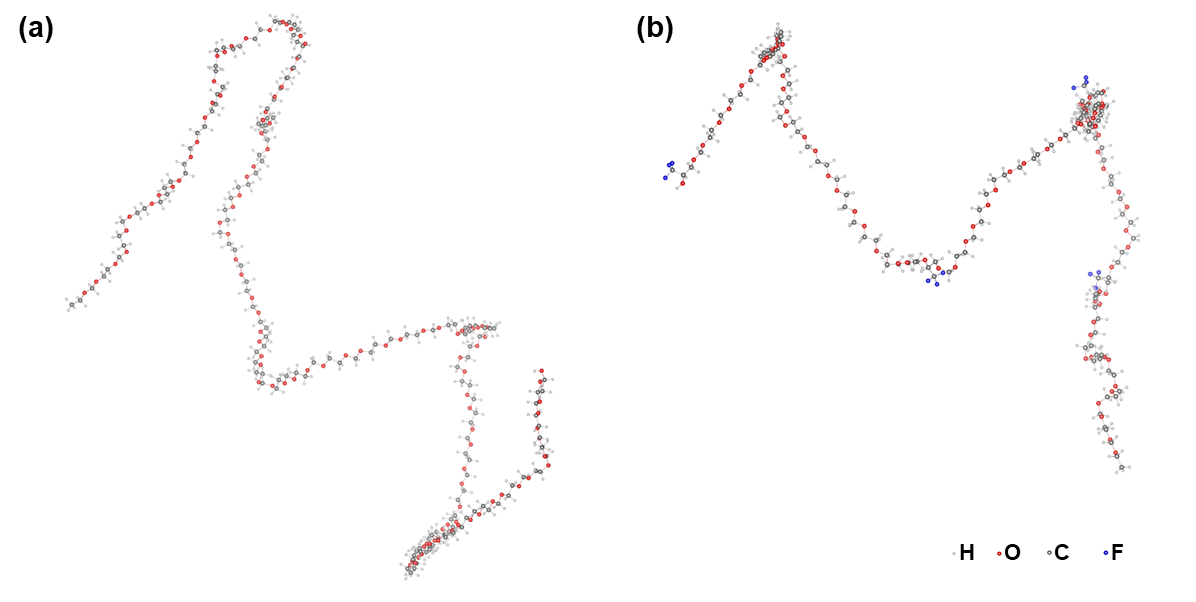


Fig. S14 Schematic representation of the molecular structure of (a) a single poly-DOL polymer chain and (b) a single poly-DOL_0.9_-TFEP_0.1_ polymer chain after geometry optimization by DFT simulation.


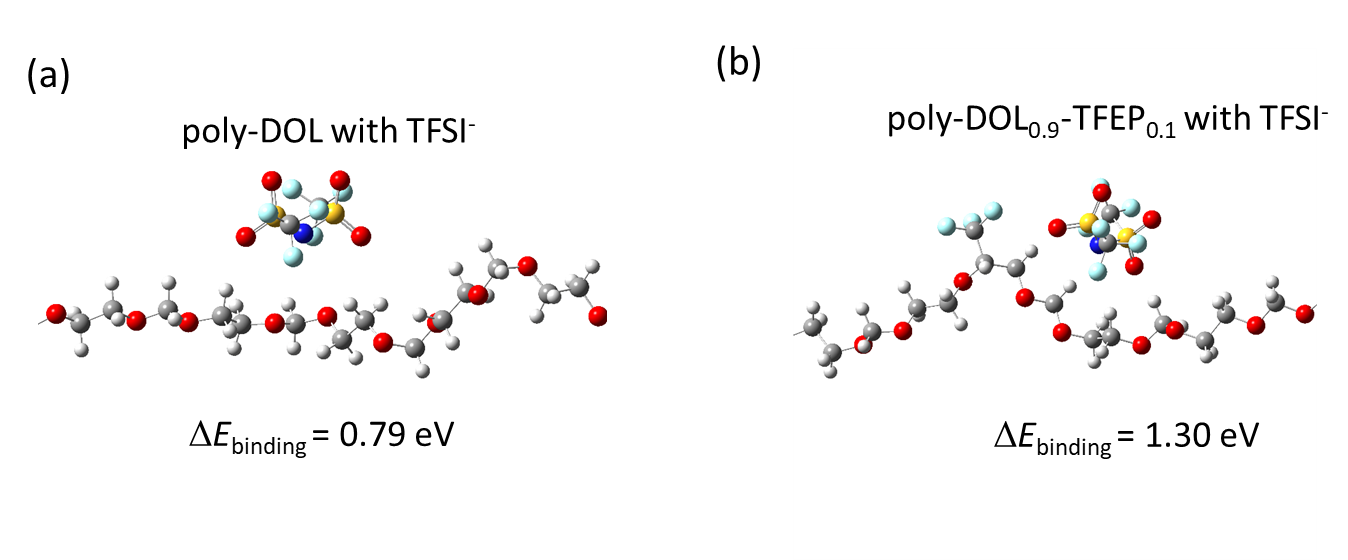


Fig. S15 The binding energy of TFSI^-^ with (a) poly-DOL polymer and poly-DOL_0.9_-TFEP_0.1_ polymer.

Note: The binding energy(*∆E*_binding_) was calculated by the following equation:

Δ*E*_binding_ = *E*_[polymer-TFSI]_^-^ - *E*_[polymer]_ - *E*_[TFSI]_^-^

where *E*_[polymer-TFSI]_^-^ represents the energy of the combination of the polymer and the TFSI^-^ anion, *E*_[polymer]_ denotes the energy of the polymer, and *E*_[TFSI]_^-^ indicates the energy of the TFSI^-^ anion.


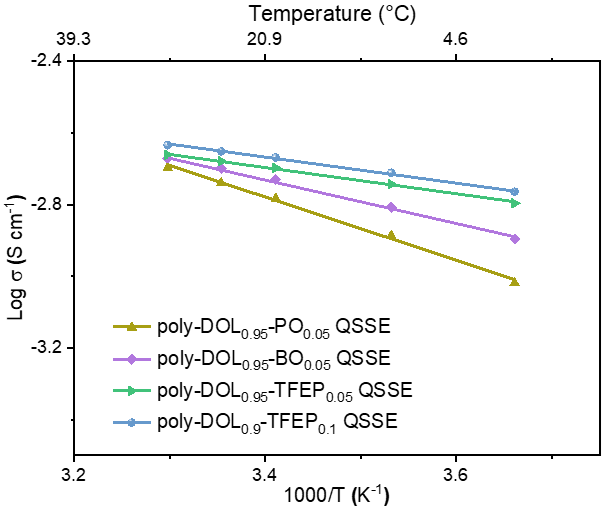


Fig. S16 The Li^+^ conductivity of poly-DOL_0_._95_-PO_0.05_ QSSE, poly-DOL_0.95_-BO_0.05_ QSSE, poly-DOL_0.95_-TFEP_0.05_ QSSE, and poly-DOL_0.9_-TFEP_0.1_ QSSE as a function of temperature.


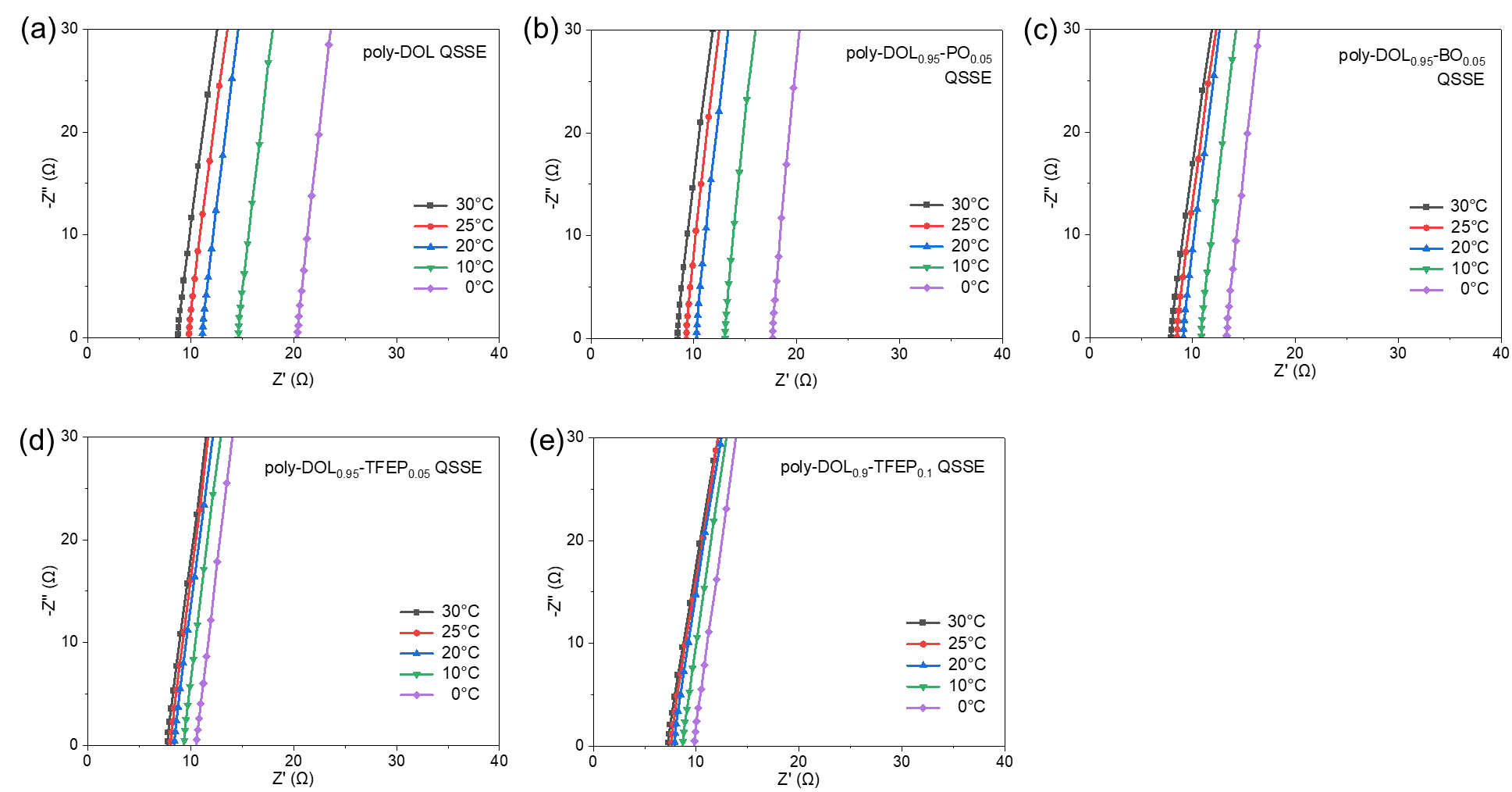


Fig. S17 Nyquist plots of (a) poly-DOL QSSE, (b) poly-DOL_0.95_-PO_0.05_ QSSE, (c) poly-DOL_0.95_-BO_0.05_ QSSE, (d) poly-DOL_0.95_-TFEP_0.05_ QSSE and (e) poly-DOL_0.9_-TFEP_0.1_ QSSE obtained with SS|electrolytes|SS cell at various temperatures.


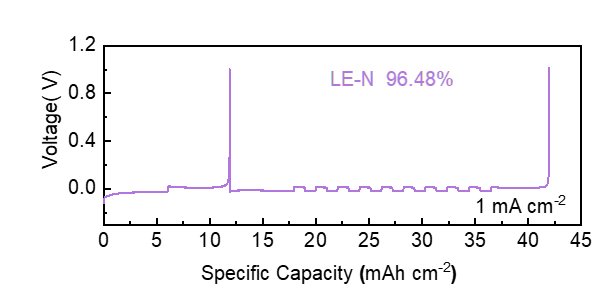


Fig. S18 Coulombic efficiencies of Li plating/stripping in Li|Cu batteries with the LE-N electrolytes.


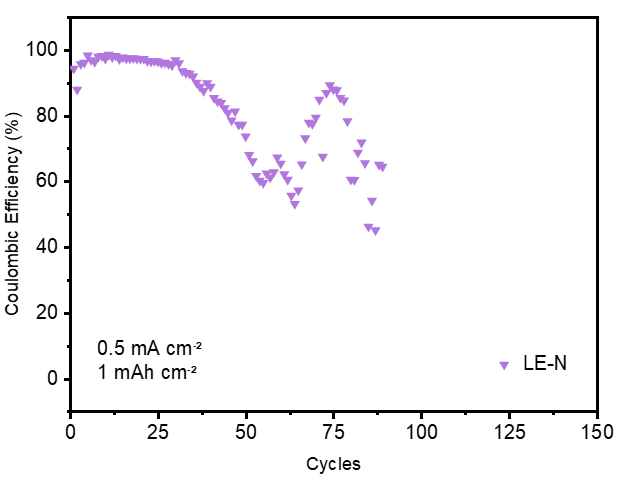


Fig. S19 Cycling performance of Li|Cu cells of the LE-N.


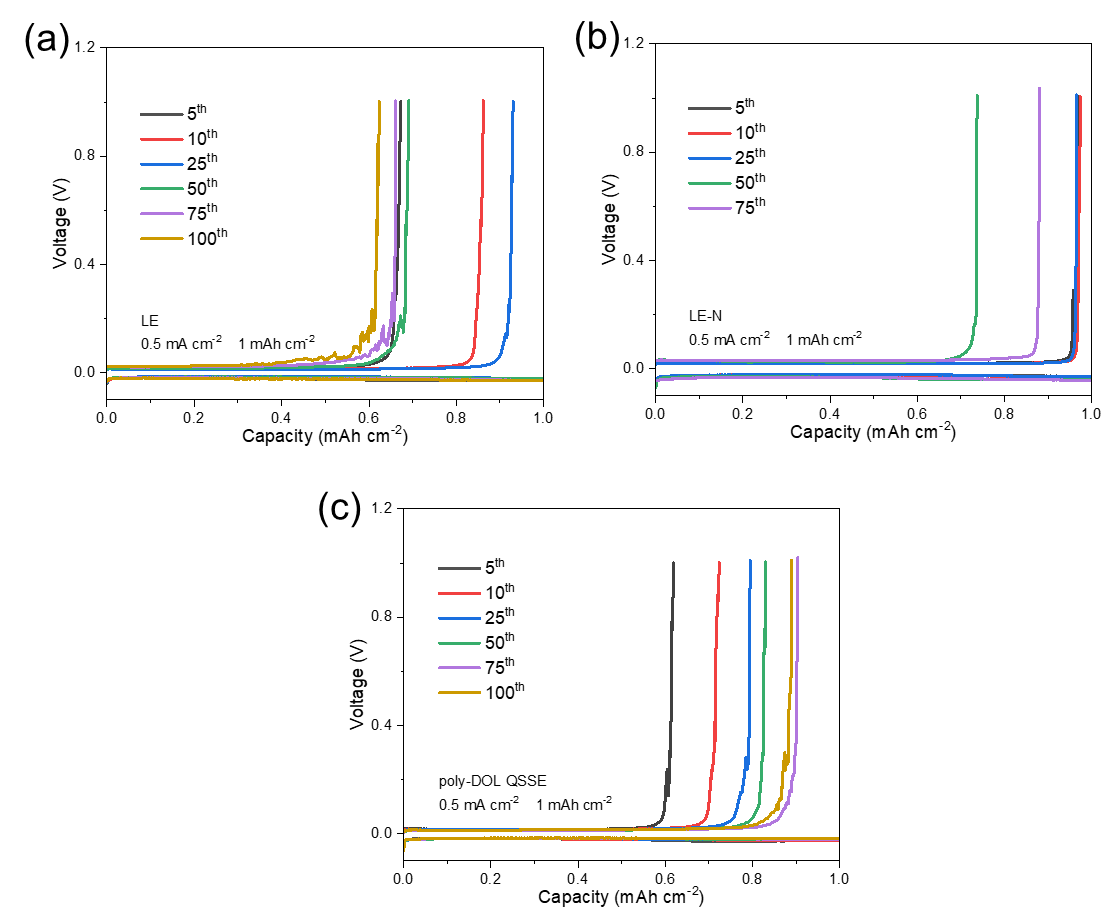


Fig. S20 Typical charge & discharge curves of the Li|Cu cells with (a) the LE, (b) the LE-N and (c) the poly-DOL QSSE.


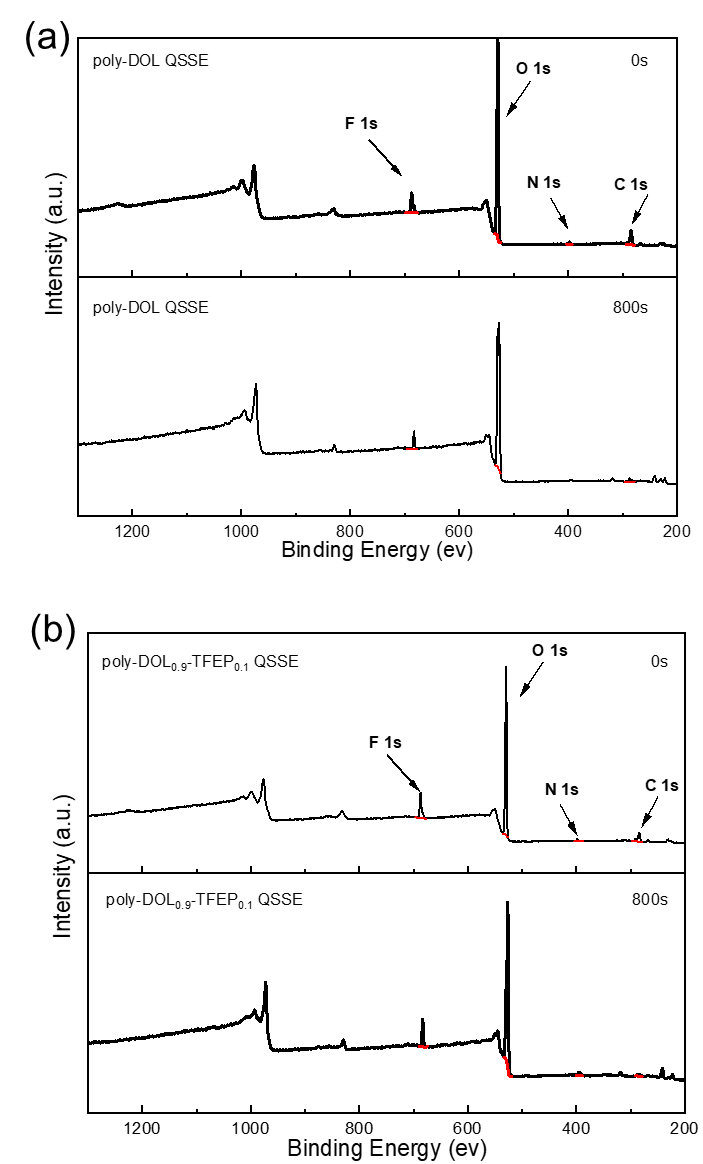


Fig. S21 XPS sum spectra of the LMA from (a) the poly-DOL QSSE battery and (b) the poly-DOL_0.9_-TFEP_0.1_ QSSE battery at various etching times.

Note: For these experiments, the LMAs were taken from Li|LiFePO_4_ batteries that cycled for 100 times.


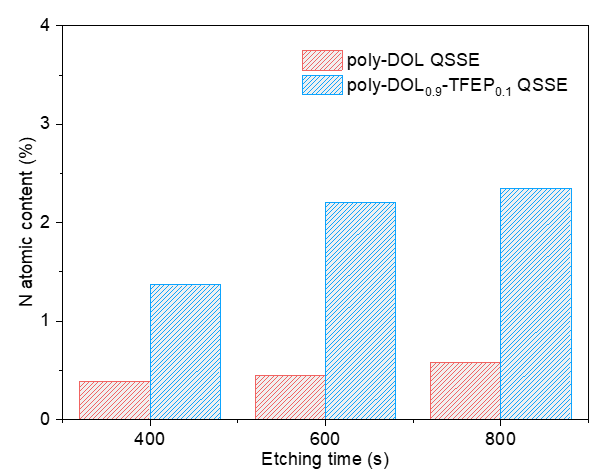


Fig. S22 XPS analysis of LMAs: nitrogen content at various etching times.

Note: For these experiments, the LMAs were taken from Li|LiFePO_4_ batteries that cycled for 100 times.


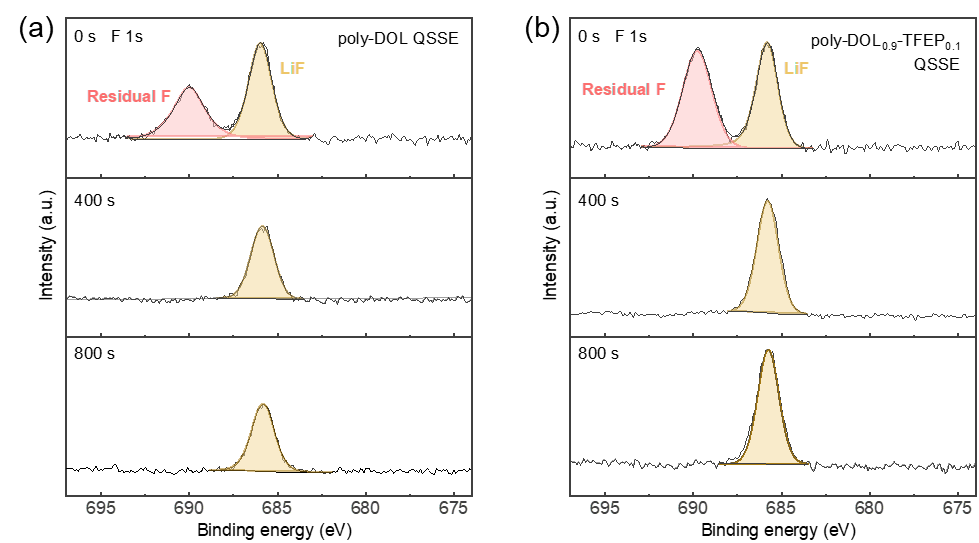


Fig. S23 F 1s XPS spectra of the LMA from (a) the poly-DOL QSSE battery and (b) the poly-DOL_0.9_-TFEP_0.1_ QSSE battery at various etching times.

Note: For these experiments, the LMAs were taken from Li|LiFePO_4_ batteries that cycled for 100 times.

.


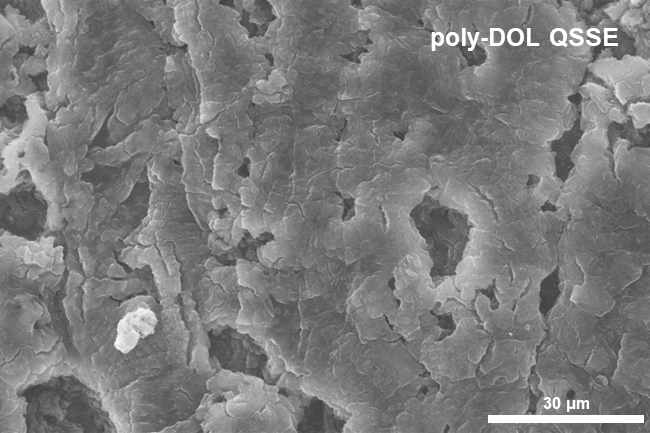


Fig. S24 SEM images of the LMA from the poly-DOL QSSE battery after 100 cycles in Li|LiFePO_4_ batteries.


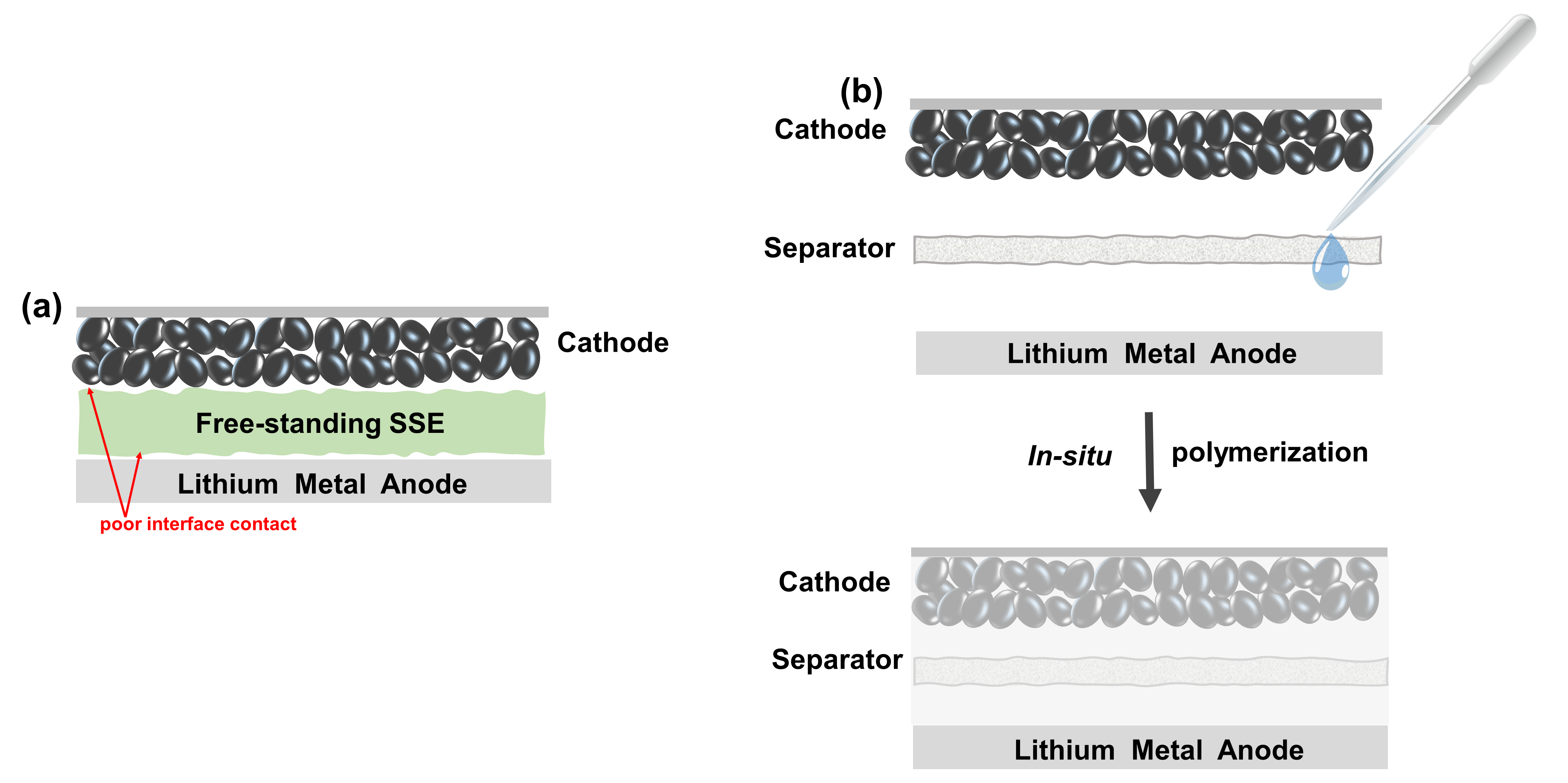


Fig. S25 (a)Schematic illustration showing poor interfacial contact in free-standing SSEs (b)Schematic illustration of integrated coin-type battery production *via* *in-situ* polymerization. Excellent interfacial contact can be obtained by *in situ* polymerization process.


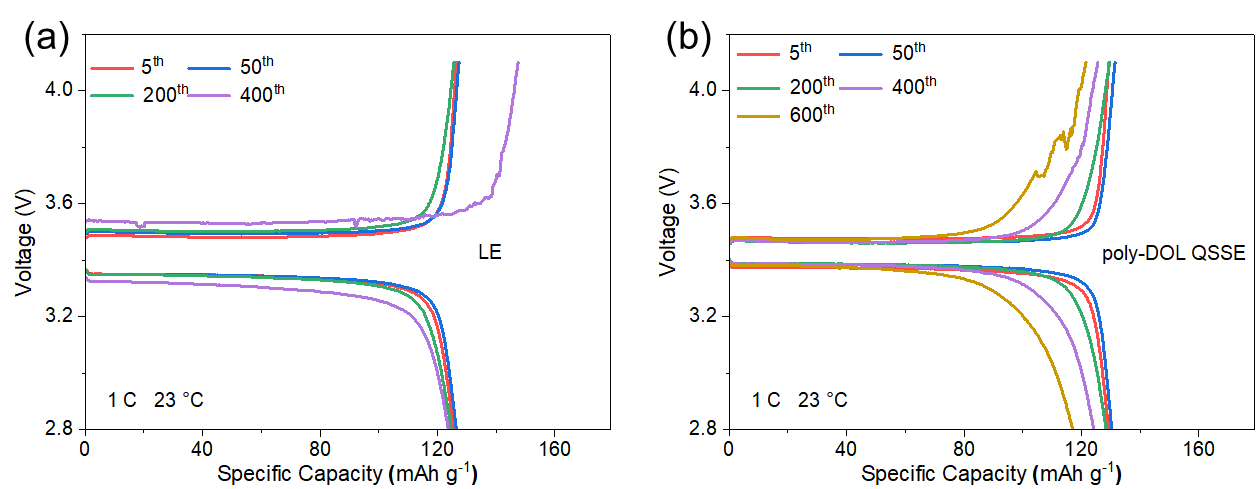


Fig. S26 Charge and discharge curves of (a) the Li|LE|LiFePO_4_ battery and (b) the Li|poly-DOL QSSE|LiFePO_4_ battery.

Note: For the Li| LiFePO_4_ battery, the liquid electrolyte (LE) employed was a 1 M LiTFSI in a 7:3 (v/v) mixture of DOL and DME. The addition of DME was to address the limited oxidation stability during cycling in LFP battery. Unless otherwise indicated, all other LE formulations mentioned in this work refer to the 1 M LiTFSI in DOL electrolyte.


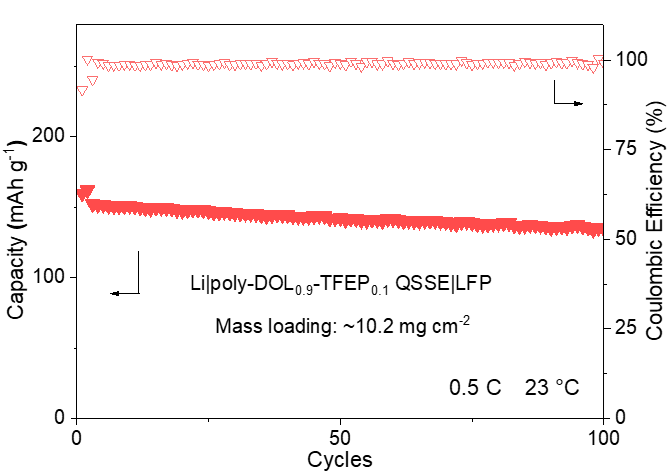


Fig. S27 Cycle performance (0.5 C) of the high loading (10.2 mg cm^-2^ for LiFePO_4_) Li| poly-DOL_0.9_-TFEP_0.1_ QSSE|LiFePO_4_ coin-type battery. The charge & discharge rates were set at 0.1 C for the first two cycles, and 0.5 C from the 3^rd^ cycle onwards.


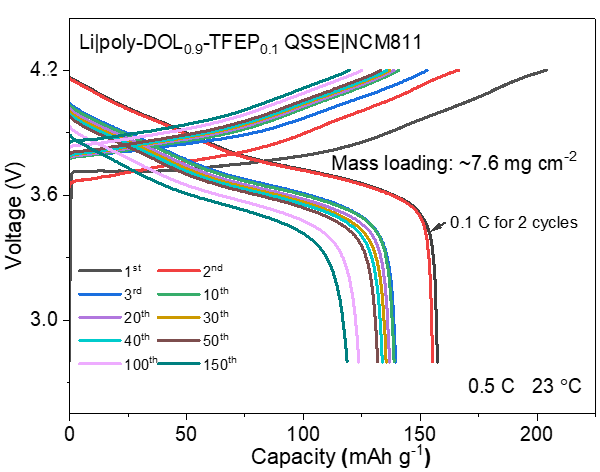


Fig. S28 Charge and discharge curves of high loading (7.6 mg cm^-2^ for NCM811) Li|poly-DOL_0.9_-TFEP_0.1_ QSSE|NCM811 cell. The charge and discharge rates were set at 0.1 C for the first two cycles and 0.5 C from the 3^rd^ cycle onward.


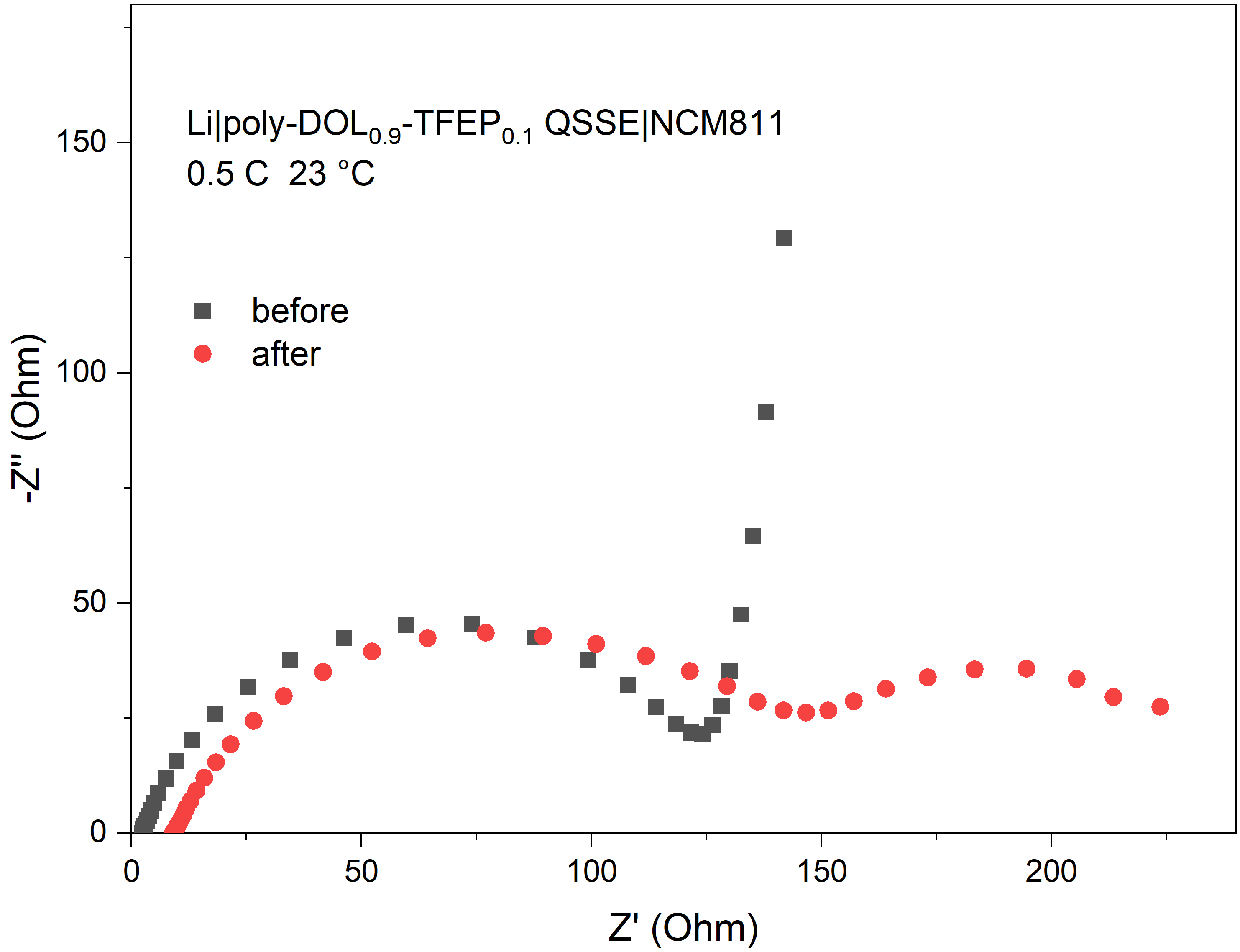


Fig. S29 Nyquist plots of the Li|poly-DOL_0.9_-TFEP_0.1_ QSSE|NCM811 cell before cycling and after 150 cycles.


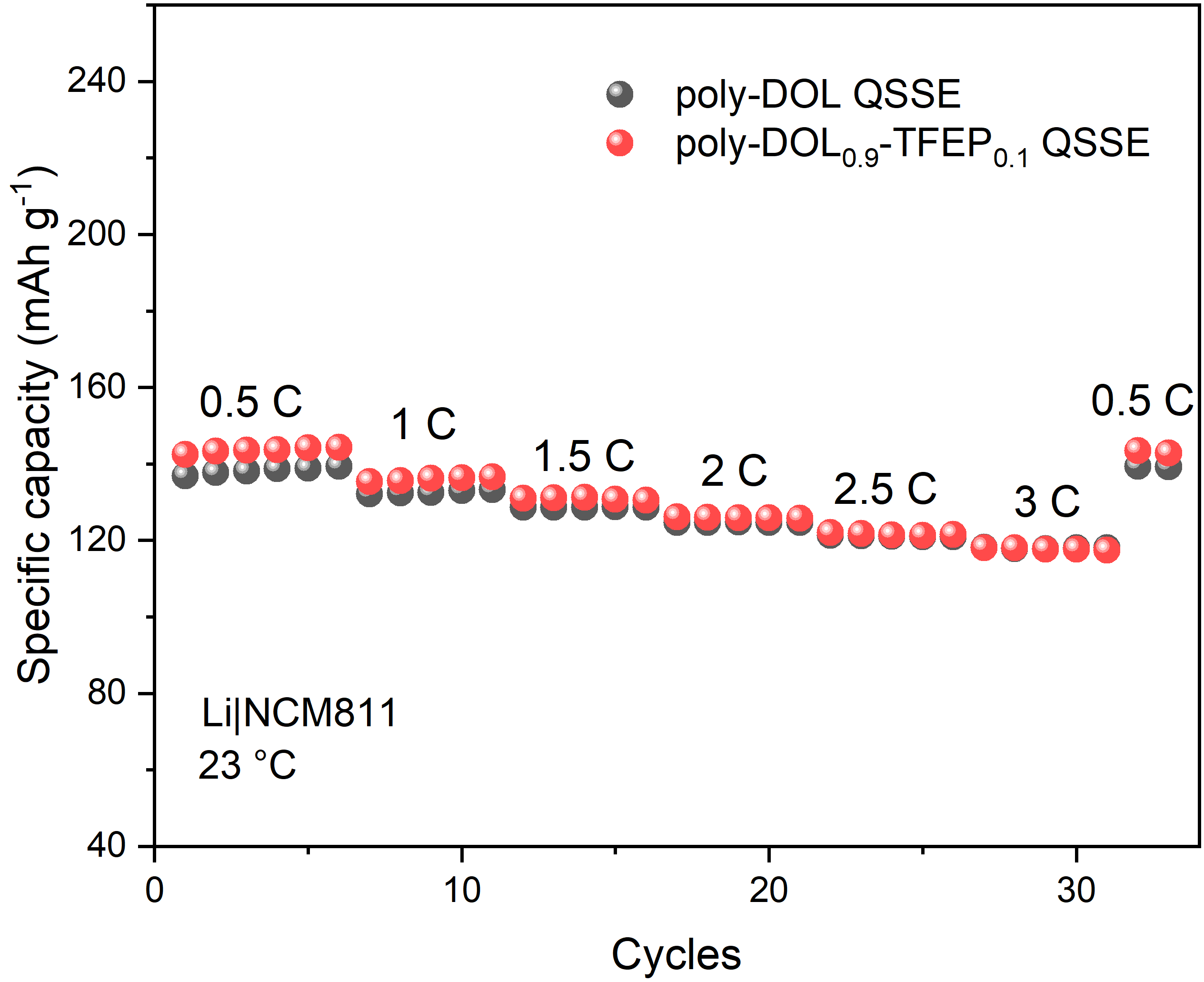


Fig. S30 Rate performance of the Li|NCM811 cells using poly-DOL QSSE and poly-DOL_0.9_-TFEP_0.1_ QSSE.


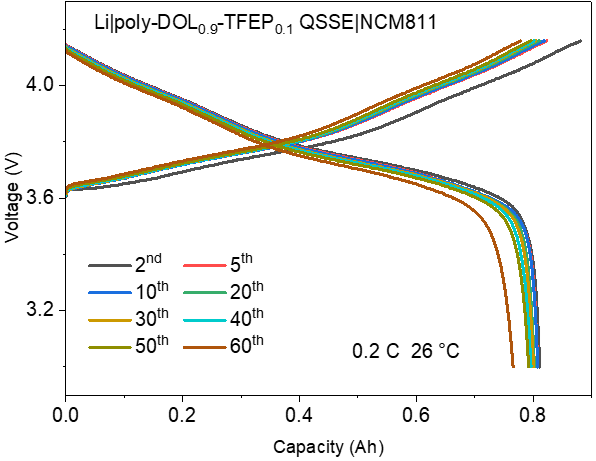


Fig. S31 Charge & discharge curves of pouch-type Li|poly-DOL_0.9_-TFEP_0.1_ QSSE|NCM811 cell. Cell was cycled at 0.1C charge / 0.2C discharge after one formation cycle at 0.05C charge / discharge.


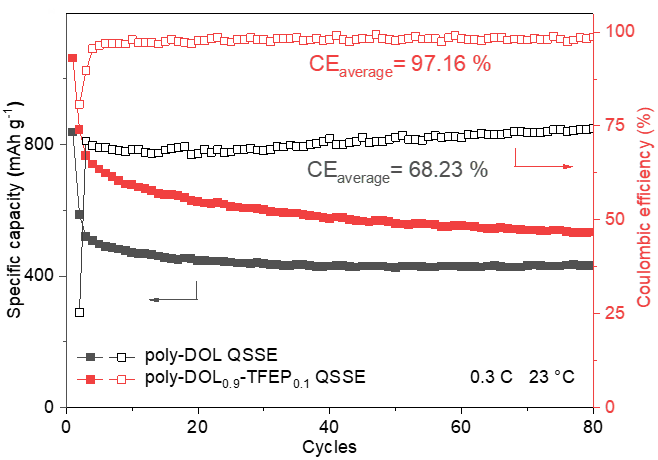


Fig. S32 Coin-type Li-S battery of poly-DOL QSSE and poly-DOL_0.9_-TFEP_0.1_ QSSE at 0.3 C (charge and discharge).

Table S1 | Polymerization enthalpies and boiling points of various cyclic ethers.

| Name | Molecular formula | -∆*H*_poly_ (kJ mol^-1^) | Boiling point(°C) |
| --- | --- | --- | --- |
| EO | 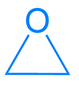 | 109.12 | 10.7 |
| PO | 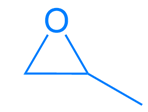 | 102.33 | 34 |
| BO | 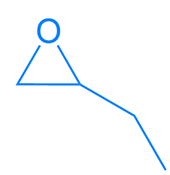 | 97.61 | 65 |
| TFEP | 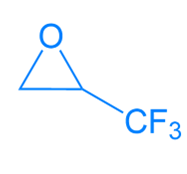 | 101.38 | 38 |
| THF | 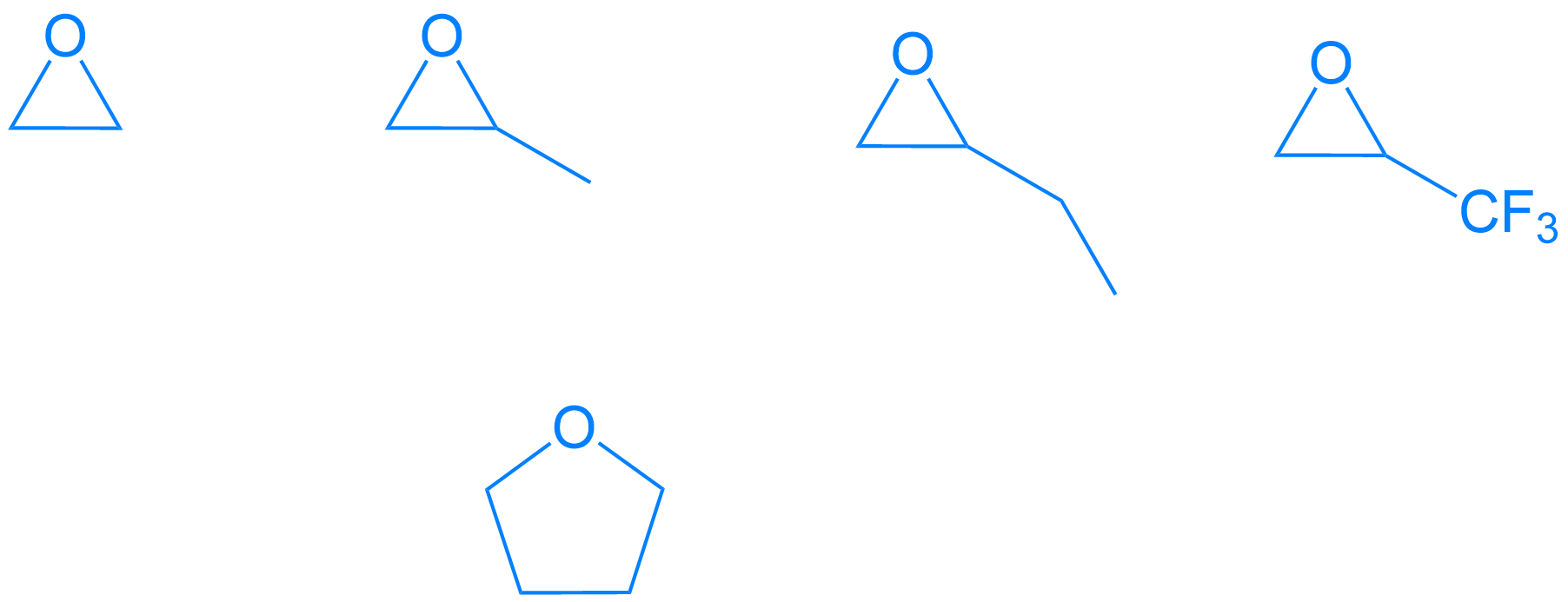 | 20.64 | 66 |
| DOL | 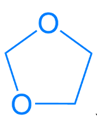 | 18.34 | 74 |

***The ΔH_poly_ and the driving force in ROP of cyclic ethers***

The thermodynamics of polymerization are governed by the Gibbs free energy (Δ*G*) of the polymerization reaction, described by the equation Δ*G*_poly_=Δ*H*_poly_-TΔ*S*_poly_. Where Δ*H*_poly_ and Δ*S*_poly_ are the differences of energies and entropies between the polymer products and their monomer reactants in the polymerization reaction, respectively, and T is the absolute temperature^[19]^. For the ROP of small cyclic monomers, typically those with six or fewer ring members, both Δ*H_poly_* and Δ*S*_poly_ are negative, and the −TΔ*S*_poly_ values usually range from 5 kJ mol^-1^ to 20 kJ mol^-1^ at ambient temperatures^[20]^. As a result, the driving force of polymerization reactions in these systems is Δ*H*_poly_, which is directly related to the intrinsic ring strain within the monomer (see Fig. S2 for more details). Monomers with higher polymerization enthalpy (refer to higher exothermic enthalpy values) tend to ROP more readily, providing a stronger thermodynamic driving force for the conversion of monomers to polymer^[19, 21]^. The Δ*H*_poly_ was calculated by following equation:

Δ*H*_poly_ = *H*_[polymer]_ - *H*_[monomers]_

Where the *H*_[polymer]_ is the enthalpy of the polymer and the *H*_[monomers]_ is the enthalpy of the monomers.

Table S2 | Polymerization period of DOL with different ratios of PO, BO and TFEP

| Name | Ratio of promoter (wt%) | Polymerization times |
| --- | --- | --- |
| DOL | 0 | Not polymerized after 5 months |
| DOL_0.95_-PO_0.05_ | 5 | ~48 hours |
| DOL_0.95_-BO_0.05_ | 5 | ~48 hours |
| DOL_0.95_-TFEP_0.05_ | 5 | ~14 days |
| DOL_0.9_-TFEP_0.1_ | 10 | ~7 days |
| DOL_0.8_-TFEP_0.2_ | 20 | ~6 days |

Note: All samples included 1M LiTFSI and 0.2 M LiNO_3_. Steel-steel symmetrical batteries were assembled using the precursor solution prepared above, and the conductivities were measured every 6-8 hours. Simultaneously, the state of the remaining precursor solutions in the vials were monitored. The polymerization is considered complete when the residual precursor solution no longer exhibited fluidity and the conductivities were stable. The polymerization kinetics of TFEP exhibit significantly longer reaction periods compared to that of PO and BO, despite the similar ∆*H*_poly_ values (Table S1). This unexpected delay can be attributed to the steric hindrance imposed by TFEP's bulky –CF_3_ substituent, which presents greater spatial constraints than the smaller –CH_3_ groups in PO and BO. Consequently, this increased steric bulk restricts monomer access to the active chain growth site, thus reducing collision frequency and slowing the overall polymerization process^[16b]^.

Table S3 | Gel permeation chromatography (GPC) results of QSSEs.

| Sample name | Number-average molecular weight (*M_n_*) |
| --- | --- |
| Poly-DOL | 4071 |
| Poly-DOL_0.9_-TFEP_0.1_ | 3427 |

Note: The samples were washed with ethanol to eliminate residual solvents and lithium salts before measurement. The number-average molecular weight suggests that a typical poly-DOL_0.9_-TFEP_0.1_ single chain is comprised, on average, of 40 DOL monomers and 4 TFEP monomers. Meanwhile, the average poly-DOL single chain consists of 55 DOL monomers. These data were utilized to construct the model for MD simulations.

Table S4 | Mole percentage of solid-state content in QSSEs.

| Sample name | Percentage of solid-state content (mol%) |
| --- | --- |
| Poly-DOL | 86.2 |
| Poly-DOL_0.9_-TFEP_0.1_ | 85.4 |

Note: The solid-state content percentage was determined through a quantitative analysis of the ^1^H NMR spectrum. This method involved calculating the relative integrated areas of the respective peaks in the spectrum. In this analysis, it is assumed that TFEP within the poly-DOL_0.9_-TFEP_0.1_ copolymer is fully incorporated into the polymer backbone during the copolymerization process. These data were utilized to construct the model for MD simulations.

Table S5| Complex modulus (G*) of poly-DOL QSSE and poly-DOL_0.9_-TFEP_0.1_ QSSE.

| Sample name | Complex modulus (G*, Pa) |
| --- | --- |
| Poly-DOL | 5690 |
| Poly-DOL_0.9_-TFEP_0.1_ | 3650 |

Table S6 | The specifications of the comparison of different Li | LiFePO_4_ coin cell with either modified N-rich SEI or poly-DOL based QSSE.

|  | Specificities | Cycles | Capacity retention (%) |
| --- | --- | --- | --- |
| **This work** | Poly-DOL-based QSSE with LiNO_3_ | 1000 | ~92.97 |
| **This work** | Poly-DOL-based QSSE with LiNO_3_ | 1500 | ~88.59 |
| Ref. A^[22]^ | Poly-DOL based QSSE | 300 | ~88.5 |
| Ref. B^[22]^ | Poly-DOL based QSSE | 600 | ~92.1 |
| Ref. C^[23]^ | Poly-DOL based QSSE | 700 | ~76 |
| Ref. D^[24]^ | Poly-DOL based QSSE | 900 | ~81.4 |
| Ref. E^[25]^ | Poly-DOL based QSSE | 400 | ~90 |
| Ref. F^[26]^ | Poly-DOL based QSSE | 550 | ~83 |
| Ref. G^[27]^ | Poly-DOL based QSSE | 200 | ~91.9 |
| Ref. H^[28]^ | Poly-DOL based QSSE | 1000 | ~83 |
| Ref. I^[29]^ | Poly-DOL based QSSE | 1000 | ~85 |
| Ref. J^[30]^ | Poly-DOL based QSSE | 500 | ~80 |
| Ref. K^[31]^ | N-rich SEI | 200 | ~94 |
| Ref. L^[32]^ | N-rich SEI | 1000 | ~82 |
| Ref. M^[33]^ | N-rich SEI | 500 | ~86.7 |
| Ref. N^[34]^ | N-rich SEI | 650 | ~88.8 |
| Ref. O^[35]^ | N-rich SEI | 120 | ~73.2 |
| Ref. P^[36]^ | N-rich SEI | 500 | ~90 |
| Ref. Q^[37]^ | N-rich SEI | 500 | ~88.9 |
| Ref. R^[38]^ | N-rich SEI | 800 | ~82 |
| Ref. S^[39]^ | N-rich SEI | 800 | ~80 |
| Ref. T^[40]^ | N-rich SEI | 1000 | ~84 |

Table S7 | The specifications of the Li | NCM811 pouch cell.

| Cell component | Specification | Parameters |
| --- | --- | --- |
| Cathode (NCM811) | Active material mass loading (mg cm^−2^, each side) | 11.27 |
|  | Active material content (%) | 96 |
|  | Number | 7 |
|  | Foil size (mm×mm) | 52×59 |
|  | Specific areal capacity (mAh cm^−2^, each side) | 1.89 |
|  | Weight (with Al foils) (g) | 5.68 |
| Anode (Li) | Thickness (μm, each side) | 20 |
|  | Area capacity (mAh cm^-2^) | ~4 |
|  | Number | 8 |
| Electrolyte | Electrolyte/Capacity (g Ah^−1^) | 2.71 |
|  | Weight (g) | 2.2 |
| Separator | Weight (with tapes) (g) | 0.78 |
| Full cell | N/P | ~2.11 |
|  | Discharge capacity (Ah) | 0.8112 |
|  | Discharge energy (Wh) | 3.09 |
|  | Total weight without Package and Tabs (g) | 10.21 |
|  | Total weight (g) | 11.95 |
|  | Specific energy without Package and Tabs (Wh kg^−1^) | 302.6 |
|  | Specific energy with Package and Tabs (Wh kg^−1^) | 258.6 |

Note: The specific energy is most accurately calculated as: specific energy = discharge energy (Wh) / total weight (kg). This method is more precise than using: specific energy = discharge capacity (Ah) × mid-value voltage (V) / total weight (kg).

**Reference**

[1] J. Yu, X. Lin, J. Liu, J. T. T. Yu, M. J. Robson, G. Zhou, H. M. Law, H. Wang, B. Z. Tang, F. Ciucci, Advanced Energy Materials 2022, 12, 2102932.

[2] M. J. Abraham, T. Murtola, R. Schulz, S. Páll, J. C. Smith, B. Hess, E. Lindahl, SoftwareX 2015, 1-2, 19.

[3] W. L. Jorgensen, D. S. Maxwell, J. Tirado-Rives, Journal of the American Chemical Society 1996, 118, 11225.

[4] a)T. Lu, F. Chen, Journal of Computational Chemistry 2012, 33, 580; b)T. Lu, F.-W. Chen, Acta Physico-Chimica Sinica 2012, 28, 1.

[5] L. Martínez, R. Andrade, E. G. Birgin, J. M. Martínez, Journal of Computational Chemistry 2009, 30, 2157.

[6] G. Bussi, D. Donadio, M. Parrinello, The Journal of Chemical Physics 2007, 126, 014101.

[7] a)B. Hess, Journal of Chemical Theory and Computation 2008, 4, 116; b)B. Hess, H. Bekker, H. J. C. Berendsen, J. G. E. M. Fraaije, Journal of Computational Chemistry 1997, 18, 1463; c)U. Essmann, L. Perera, M. L. Berkowitz, T. Darden, H. Lee, L. G. Pedersen, The Journal of Chemical Physics 1995, 103, 8577; d)T. Darden, D. York, L. Pedersen, The Journal of Chemical Physics 1993, 98, 10089.

[8] M. J. Frisch, G. W. Trucks, H. B. Schlegel, G. E. Scuseria, M. A. Robb, J. R. Cheeseman, G. Scalmani, V. Barone, G. A. Petersson, H. Nakatsuji, X. Li, M. Caricato, A. V. Marenich, J. Bloino, B. G. Janesko, R. Gomperts, B. Mennucci, H. P. Hratchian, J. V. Ortiz, A. F. Izmaylov, J. L. Sonnenberg, Williams, F. Ding, F. Lipparini, F. Egidi, J. Goings, B. Peng, A. Petrone, T. Henderson, D. Ranasinghe, V. G. Zakrzewski, J. Gao, N. Rega, G. Zheng, W. Liang, M. Hada, M. Ehara, K. Toyota, R. Fukuda, J. Hasegawa, M. Ishida, T. Nakajima, Y. Honda, O. Kitao, H. Nakai, T. Vreven, K. Throssell, J. A. Montgomery Jr., J. E. Peralta, F. Ogliaro, M. J. Bearpark, J. J. Heyd, E. N. Brothers, K. N. Kudin, V. N. Staroverov, T. A. Keith, R. Kobayashi, J. Normand, K. Raghavachari, A. P. Rendell, J. C. Burant, S. S. Iyengar, J. Tomasi, M. Cossi, J. M. Millam, M. Klene, C. Adamo, R. Cammi, J. W. Ochterski, R. L. Martin, K. Morokuma, O. Farkas, J. B. Foresman, D. J. Fox, Wallingford, CT 2016.

[9] P. J. Stephens, F. J. Devlin, C. F. Chabalowski, M. J. Frisch, The Journal of Physical Chemistry 1994, 98, 11623.

[10] a)W. J. Hehre, R. Ditchfield, J. A. Pople, The Journal of Chemical Physics 1972, 56, 2257; b)P. C. Hariharan, J. A. Pople, Theoretica chimica acta 1973, 28, 213.

[11] a)D. Feller, Journal of Computational Chemistry 1996, 17, 1571; b)T. H. Dunning, Jr., The Journal of Chemical Physics 1989, 90, 1007.

[12] a)F. Neese, WIREs Computational Molecular Science 2012, 2, 73; b)F. Neese, F. Wennmohs, U. Becker, C. Riplinger, The Journal of Chemical Physics 2020, 152, 224108.

[13] a)S. Grimme, S. Ehrlich, L. Goerigk, Journal of Computational Chemistry 2011, 32, 1456; b)E. Caldeweyher, S. Ehlert, A. Hansen, H. Neugebauer, S. Spicher, C. Bannwarth, S. Grimme, The Journal of Chemical Physics 2019, 150, 154122.

[14] F. Weigend, R. Ahlrichs, Physical Chemistry Chemical Physics 2005, 7, 3297.

[15] M. D. Hanwell, D. E. Curtis, D. C. Lonie, T. Vandermeersch, E. Zurek, G. R. Hutchison, Journal of Cheminformatics 2012, 4, 17.

[16] a)P. Kubisa, J. P. Vairon, in Polymer Science: A Comprehensive Reference, (Eds: K. Matyjaszewski, M. Möller), Elsevier, Amsterdam 2012; b)in Principles of Polymerization, 2004.

[17] S. Penczek, J. Pretula, S. Slomkowski, 2021, 3, 33.

[18] a)M. Bednarek, P. Kubisa, Macromolecular Symposia 1998, 132, 349; b)M. Bednarek, T. Biedron, P. Kubisa, S. Penczek, Makromolekulare Chemie. Macromolecular Symposia 1991, 42-43, 475.

[19] S. C. Greer, The Journal of Physical Chemistry B 1998, 102, 5413.

[20] A. Duda, A. Kowalski, in Handbook of Ring‐Opening Polymerization, 2009.

[21] H. Tran, A. Toland, K. Stellmach, M. K. Paul, W. Gutekunst, R. Ramprasad, The Journal of Physical Chemistry Letters 2022, 13, 4778.

[22] K. Mu, D. Wang, W. Dong, Q. Liu, Z. Song, W. Xu, P. Yao, Y. a. Chen, B. Yang, C. Li, L. Tian, C. Zhu, J. Xu, Advanced Materials 2023, 35, 2304686.

[23] Q. Zhao, X. Liu, S. Stalin, K. Khan, L. A. Archer, Nature Energy 2019, 4, 365.

[24] Z. Ren, J. Li, M. Cai, R. Yin, J. Liang, Q. Zhang, C. He, X. Jiang, X. Ren, Journal of Materials Chemistry A 2023, 11, 1966.

[25] F. Liu, T. Li, Y. Yang, J. Yan, N. Li, J. Xue, H. Huo, J. Zhou, L. Li, Macromolecular Rapid Communications 2020, 41, 2000047.

[26] J. Yu, G. Zhou, Y. Li, Y. Wang, D. Chen, F. Ciucci, Small 2023, 19, 2302691.

[27] T. Yang, W. Zhang, Y. Liu, J. Zheng, Y. Xia, X. Tao, Y. Wang, X. Xia, H. Huang, Y. Gan, X. He, J. Zhang, Small 2023, 19, 2303210.

[28] J. Ma, Y. Wu, H. Jiang, X. Yao, F. Zhang, X. Hou, X. Feng, H. Xiang, ENERGY & ENVIRONMENTAL MATERIALS 2023, 6, e12370.

[29] Q. Liu, B. Cai, S. Li, Q. Yu, F. Lv, F. Kang, Q. Wang, B. Li, Journal of Materials Chemistry A 2020, 8, 7197.

[30] X. Yang, D. Ye, C. Wang, Y. Chen, X. Jiang, Y. Yang, Z. Liu, Journal of Power Sources 2024, 600, 234262.

[31] Y. Wen, J. Ding, Y. Yang, X. Lan, J. Liu, R. Hu, M. Zhu, Advanced Functional Materials 2022, 32, 2109377.

[32] Z. Hu, Y. Wang, S. Huo, W. Bao, W. Fan, Y. Zhang, X. Jing, N. Ahmad, H. Cheng, Y. Zhang, Journal of Membrane Science 2023, 677, 121643.

[33] X. Zeng, W. Wang, H. Hu, W. Fan, C. Fan, J. Nan, Electrochimica Acta 2022, 414, 140212.

[34] X. Weng, Y. Qin, X. Da, Y. Zhao, X. Deng, B. Wen, M. Cui, X. Yin, Y. Su, J. Song, S. Ding, X. Hu, G. Gao, X. Li, Chemical Engineering Journal 2023, 466, 143302.

[35] S. Kim, K.-Y. Cho, J. Kwon, K. Sim, D. Seok, H. Tak, J. Jo, K. Eom, Small 2023, 19, 2207222.

[36] J. Pokharel, A. Cresce, B. Pant, M. Y. Yang, A. Gurung, W. He, A. Baniya, B. S. Lamsal, Z. Yang, S. Gent, X. Xian, Y. Cao, W. A. Goddard, K. Xu, Y. Zhou, Nature Communications 2024, 15, 3085.

[37] S. Zhang, H. Liu, Z. Liu, Y. Zhao, J. Yan, Y. Zhang, F. Liu, Q. Liu, C. Liu, G. Sun, Z. Wang, J. Yang, Y. Ren, Advanced Functional Materials 2024, n/a, 2401377.

[38] J. Jiang, M. Li, X. Liu, J. Yi, Y. Jiang, C. Wu, H. Liu, B. Zhao, W. Li, X. Sun, J. Zhang, S. Dou, Advanced Energy Materials 2024, n/a, 2400365.

[39] X. Pei, Y. Li, T. Ou, X. Liang, Y. Yang, E. Jia, Y. Tan, S. Guo, Angewandte Chemie International Edition 2022, 61, e202205075.

[40] Y. Li, P. Huang, C. Cui, X. Ma, L. Chen, Z. Li, L. Li, Y. Tian, Progress in Natural Science: Materials International 2024, 34, 338.
